# Unveiling Kiwifruit Metabolite and Protein Changes in the Course of Postharvest Cold Storage

**DOI:** 10.3389/fpls.2019.00071

**Published:** 2019-02-04

**Authors:** Anna Maria Salzano, Giovanni Renzone, Anatoly P. Sobolev, Virginia Carbone, Milena Petriccione, Donatella Capitani, Monica Vitale, Gianfranco Novi, Nicola Zambrano, Maria Silvia Pasquariello, Luisa Mannina, Andrea Scaloni

**Affiliations:** ^1^Proteomics & Mass Spectrometry Laboratory, Istituto per il Sistema Produzione Animale In Ambiente Mediterraneo, National Research Council, Naples, Italy; ^2^Magnetic Resonance Laboratory “Annalaura Segre”, Institute of Chemical Methodologies, National Research Council, Monterotondo, Italy; ^3^Institute of Food Sciences, National Research Council, Avellino, Italy; ^4^Centro di Ricerca per Olivicoltura, Frutticoltura e Agrumicoltura, Consiglio per la Ricerca in Agricoltura e l’Analisi dell’Economia Agraria, Caserta, Italy; ^5^Dipartimento di Medicina Molecolare e Biotecnologie Mediche, Università degli Studi di Napoli Federico II, Naples, Italy; ^6^Ceinge Biotecnologie Avanzate S. C. a R. L., Naples, Italy; ^7^Dipartimento di Chimica e Tecnologie del Farmaco, Sapienza Università di Roma, Rome, Italy

**Keywords:** kiwifruit, postharvest, metabolomics, proteomics, cold storage

## Abstract

*Actinidia deliciosa* cv. Hayward fruit is renowned for its micro- and macronutrients, which vary in their levels during berry physiological development and postharvest processing. In this context, we have recently described metabolic pathways/molecular effectors in fruit outer endocarp characterizing the different stages of berry physiological maturation. Here, we report on the kiwifruit postharvest phase through an integrated approach consisting of pomological analysis combined with NMR/LC-UV/ESI-IT-MS^n^- and 2D-DIGE/nanoLC-ESI-LIT-MS/MS-based proteometabolomic measurements. Kiwifruit samples stored under conventional, cold-based postharvest conditions not involving the use of dedicated chemicals were sampled at four stages (from fruit harvest to pre-commercialization) and analyzed in comparison for pomological features, and outer endocarp metabolite and protein content. About 42 metabolites were quantified, together with corresponding proteomic changes. Proteomics showed that proteins associated with disease/defense, energy, protein destination/storage, cell structure and metabolism functions were affected at precise fruit postharvest times, providing a justification to corresponding pomological/metabolite content characteristics. Bioinformatic analysis of variably represented proteins revealed a central network of interacting species, modulating metabolite level variations during postharvest fruit storage. Kiwifruit allergens were also quantified, demonstrating in some cases their highest levels at the fruit pre-commercialization stage. By lining up kiwifruit postharvest processing to a proteometabolomic depiction, this study integrates previous observations on metabolite and protein content in postharvest berries treated with specific chemical additives, and provides a reference framework for further studies on the optimization of fruit storage before its commercialization.

## Introduction

Green-fleshed kiwifruit (*Actinidia deliciosa*) is an economically valuable crop that is highly appreciated for its unique flavor and abundant antioxidants, dietary fiber and amino acids (Tavarini et al., 2008). Generally, fruit is gathered at a physiological ripe stage that, however, does not match to a proper palatable condition, as a result of its firmness and sourness features. Fruit ripening to an edible condition is obtained through a proper berry postharvest management by temperature and chemical treatments, which modulate ethylene (C_2_H_4_) production and peak respiration. This phase is essential in kiwifruit industry, since it reduces important economic losses due to fruit senescence and optimizes pomological characteristics of the ripe material before its commercialization (Antunes and Sfakiotakis, 2002; Antunes, 2007; Richardson et al., 2011).

It is well known that fruit ripening is regulated by C_2_H_4_ in climacteric species, while it is virtually independent of this compound in non-climacteric counterparts. In the first ones, ripening parallels a change from a negative feedback regulation (system I) to a positive one (system II) of C_2_H_4_ production (McMurchie et al., 1972), through C_2_H_4_-induced expression of 1-aminocyclopropane-1-carboxylate (ACC) synthase (ACS) and ACC oxidase (ACO) (Tucker, 1993; Lelièvre et al., 1997). C_2_H_4_ then binds to ethylene receptors and response factors regulating transcription of genes affecting the fruit ripening processes (Yin et al., 2010). Although kiwifruit is considered as a climacteric species, a number of ripening–associated changes occur before system II-dependent C_2_H_4_ is produced (Richardson et al., 2011; McAtee et al., 2015). In addition, general fruit ripening in cold storage occurs in the lack of measurable C_2_H_4_ (Hewett et al., 1999; Kim et al., 1999; Ilina et al., 2010). Indeed, a recent transcriptomic study has shown that kiwifruit has both C_2_H_4_-dependent and low temperature-modulated ripening mechanisms, which are distinct and autonomous (Asiche et al., 2018). These findings provided a foundation in evaluating the whole ripening process in this fruit.

A great challenge in postharvest management of kiwifruit is the occurrence of diseases caused by pathogenic *Botrytis cinerea*, *Botryosphaeria* sp., and *Phomopsis* sp. (Manning et al., 2016). Diseased fruit release infection-induced C_2_H_4_, which may in turn prompt ripening and feedback regulation of C_2_H_4_ production in healthy contiguous kiwifruits. In the context of postharvest management, the positive effect of treating kiwifruit by low temperatures (Günther et al., 2015; Park et al., 2015b; Minas et al., 2016), with exogenous ozone (Minas et al., 2012, 2014; Tanou et al., 2015), sodium nitroprusside (Tanou et al., 2015), 1-methylcyclopropene (Mworia et al., 2012; Park et al., 2015a; Thongkum et al., 2018), acetylsalicylic acid (Zhang et al., 2003), C_2_H_4_ (Hu et al., 2016; Minas et al., 2016; Park et al., 2016) and propylene (Asiche et al., 2016, 2018), or a combination of them (Minas et al., 2014, 2016; Tanou et al., 2015) was assessed, although the latter procedures have found a partial diffusion in kiwifruit industry due to their technology costs. Nevertheless, these studies provided important information on the effect of the application of these postharvest treatments on fruit firmness, respiration, acidity, shelf-life and decay, as well as on ethylene, soluble solid, reducing sugar, starch, antioxidant, and volatile compound content. In some cases, transcriptomic and/or proteomic investigations were also accomplished on the same fruit samples (Zhang et al., 2003; Minas et al., 2012, 2014, 2016; Mworia et al., 2012; Tanou et al., 2015; Asiche et al., 2016; Hu et al., 2016; Thongkum et al., 2018), describing differentially expressed genes and/or represented proteins in treated kiwifruits (with respect to control) that, in the latter case, were identified by MS-based procedures searching the genome of yellow-fleshed kiwifruit (*A. chinensis*) (Huang et al., 2013; Pilkington et al., 2018). Thus, very interesting proteomic studies were performed on berries subjected to treatment with specific chemical additives during postharvest storage at 0°C, which were then removed from these environments and allowed to ripe at 20°C before their proteomic evaluation (Minas et al., 2012, 2014, 2016; Tanou et al., 2015; Ainalidou et al., 2016). These differential investigations lacked information on fruits non-subjected to chemical treatments (control), and were accomplished on samples as obtained at the end of the postharvest process, missing data on the dynamics of protein quantitative changes during cold storage. In addition, they were based on gel silver staining procedures, which suffer of recognized drawbacks due to their limited accuracy in quantitative measurements; the latter were overcome with 2D-DIGE technology (Alban et al., 2003). Chromatography-based metabolomic studies on the same samples were also realized with the aim of evaluating corresponding metabolite concentration variations as result of different postharvest treatments (Günther et al., 2015; Ainalidou et al., 2016).

Proteomic and metabolomic studies reported above represented the interest of fruit industry in understanding active metabolic pathways and molecular processes associated with postharvest berry management. In particular, proteomics has widely been applied in the description of quantitative protein changes occurring during ripening and postharvest phases of apple (Costa et al., 2010; Zheng et al., 2013; Liu et al., 2016), mandarin (Yun et al., 2013), grape (Cai et al., 2014), pear (Pedreschi et al., 2008, 2009; Wang et al., 2017), banana (Li et al., 2015; Du et al., 2016; Xiao et al., 2018), papaya (Huerta-Ocampo et al., 2012; Nogueira et al., 2012), mango (Andrade et al., 2012), peach (Borsani et al., 2009; Lara et al., 2009; Nilo et al., 2010; Zhang et al., 2010, 2011, 2012; Giraldo et al., 2012; Jiang et al., 2014; Lauxmann et al., 2014; Wu et al., 2016; Tanou et al., 2017; Xi et al., 2017) and tomato (Sun et al., 2016). These studies paralleled those on berry physiological development (Palma et al., 2011; Molassiotis et al., 2013), and identified variably-represented metabolic pathways and protein effectors in these climacteric and non-climacteric fruits, rationalizing their grouping on a molecular basis, and linking these compounds to corresponding pomological characteristics, increased respiration rate and C_2_H_4_ biosynthesis.

Taking advantage of our previous experience in a NMR/LC-UV/ESI-IT-MS^n^- and 2D-DIGE/nanoLC-ESI-LIT-MS/MS-based description of kiwifruit physiological development (Salzano et al., 2018), we used the same procedure to fill the above-mentioned gaps and to describe on a time-course basis proteometabolomic changes in harvest kiwifruit stored at a low temperature, in the absence of chemical additives. Bioinformatic elaboration of resulting data suggested metabolic pathways and molecular processes/interactions affected during different postharvest moments, providing a rationale to corresponding pomological characteristics, and integrating previous proteomic observations on postharvest kiwifruit treated with specific chemicals (Minas et al., 2012, 2016; Tanou et al., 2015; Ainalidou et al., 2016).

## Materials and Methods

### Fruit Sampling and Pomological Measurements

Kiwifruit (*A. deliciosa* cv. Hayward) samples were harvested from a commercial orchard located in Francolise (Caserta, Italy). Fruits were randomly sampled from 10 selected vines at the commercial ripening stage 82 of the BBCH scale (Salinero et al., 2009); they were selected for uniformity and the absence of physical defects/decay. Healthy fruits were stored in a controlled chamber at 4°C, with 85% relative humidity, and removed after 0 (T0), 30 (T1), 60 (T2), and 90 (T3) days of cold storage. At each post-harvest stage, 60 selected fruits were sampled and divided in 3 biological replicates, which were quickly used for the measurement of pomological and qualitative traits (see Supplementary Material for details). They were also quickly peeled and their outer pericarp (without inner pericarp containing locules and seeds) was sampled, rapidly cut, frozen in liquid N_2_ and stored to -80°C, until used for further metabolomic and proteomic analyses.

### NMR Analysis of Metabolites

Extraction of metabolites from outer pericarp samples (about 2 g) taken at different postharvest stages was carried out as previously described (Salzano et al., 2018). Briefly, fruit powder samples were treated with a methanol/chloroform mixture generating corresponding hydroalcoholic and organic extracts (see Supplementary Material for details), which were then dried and stored at -20°C. Hydroalcoholic extracts were solved in 0.7 ml phosphate buffer in D_2_O containing 2 mM 3-(trimethylsilyl)-propionic-2,2,3,3-d_4_ acid sodium salt (TSP) (used as internal standard). Organic counterparts were solved in 0.7 ml of 2:1 v/v CDCl_3_/CD_3_OD. NMR spectra were recorded at 27°C on a Bruker AVANCE 600 instrument operating under experimental conditions described previously (Salzano et al., 2018) and in Supplementary Material. Assignment of ^1^H spectra of aqueous and organic extracts was achieved as previously reported (Salzano et al., 2018). Metabolite concentrations were derived from the integral values of the corresponding resonances in ^1^H NMR spectra (Supplementary Material). NMR data were subjected to PCA, which was performed using Statistica software for Windows (Statsoft, United States).

### LC-UV Analysis of Metabolites Combined With ESI-IT-MS^n^

Polyphenolic compounds from kiwifruit samples taken at T0–T3 were extracted as reported previously (Salzano et al., 2018) and in Supplementary Material. They were resolved onto C18 Sep-Pak cartridges (Waters, Milford, MA, United States) and analyzed by HPLC-UV with a HP 1110 instrument (Agilent, Palo Alto, CA, United States), monitoring absorbance at 280 nm; column and chromatographic conditions were described previously (Salzano et al., 2018). Identification of phenolic compounds present in the different HPLC fractions was carried out by ESI-IT-MS^n^ analysis using a Finnigan LCQ DECA XP Max ion trap mass spectrometer (Thermo Fisher Scientific, San Jose, CA, United States), operating as previously described (Salzano et al., 2018). For quantitative analysis, a standard curve for each polyphenol derivative was prepared by using standard compounds over a concentration range of 0.25–5 μg/μl, by means of six different concentration levels and duplicate injections at each level. All samples had three replications and each replicate was measured twice. Results were expressed as mg/kg of fresh weight (FW). Statistical analysis (one-way analysis of variance and multiple mean comparisons Tukey’s HSD) of the concentration of individual/total polyphenolic compounds was performed using SPSS Software Package, version 20.0 (SPSS Inc., Chicago, IL, United States).

### Protein Extraction and 2D-DIGE Analysis

Kiwifruit outer pericarp samples taken at T0–T3 were extracted for proteins using a modified version of the phenol/SDS-based method (Corrado et al., 2012; D’Ambrosio et al., 2013; Salzano et al., 2018), which were then quantified as reported in Supplementary Material. Dried kiwifruit proteins from 3 biological replicates of each postharvest stage (50 μg) were independently solved in 2 M thiourea, 7 M urea and 4% w/v CHAPS, and labeled with 400 pmol of Cy2-, Cy3- or Cy5-dyes (GE Healthcare) using the dye-swapping strategy (Tamburino et al., 2017). Proteins in each biological replicate were then resolved and quantified according to the 2D-DIGE procedure (Alban et al., 2003; Salzano et al., 2018) (see Supplementary Material for details). A parallel preparative 2-DE experiment was also performed using 500 μg of unlabeled proteins, which were stained with Sypro Ruby (Thermo Fisher). After spot matching with the master gel from the 2D-DIGE experiment, spots in 2-DE corresponding to those showing quantitative abundance changes in 2D-DIGE were picked with an Ettan spot robotic picker (GE Healthcare), and analyzed for protein identification.

### Protein Identification by Mass Spectrometry Analysis

2-DE spots were excised, *S*-alkylated with iodoacetamide, and digested with trypsin (Guarino et al., 2007; Scippa et al., 2010; Tamburino et al., 2017); resulting peptides were resolved on an Easy C18 column (100 mm × 0.075 mm, 3 μm) (Thermo Fisher) and analyzed under a CID-MS/MS data-dependent product ion scanning procedure with a LTQ XL mass spectrometer (Thermo Fisher), as previously reported (Scippa et al., 2010; Corrado et al., 2012; Salzano et al., 2018). Raw MS and MS/MS data were searched using MASCOT software (v. 2.2.06, Matrix Science, United Kingdom) against the *A. chinensis* protein sequence database (39,040 entries) (Huang et al., 2013), using previously reported (Scippa et al., 2010; D’Ambrosio et al., 2013; Salzano et al., 2018) and default MASCOT parameters. Protein candidates having at least 2 sequenced peptides with an individual peptide ion score >30 and a peptide expectation value <0.05 (consistent with a confidence level >95%) were considered surely identified. Protein assignment was always associated with manual verification. Identified proteins were further filtered according to an EMPAI ratio criterion (EMPAI 1st/EMPAI 2nd > 2). Proteomic data have been deposited to the ProteomeXchange Consortium (Vizcaíno et al., 2016) via the PRIDE partner repository with the dataset identifier PXD011949.

### Bioinformatics

Identified proteins were subjected to BLAST sequence homology search against the *Arabidopsis thaliana* protein sequence database TAIR 10 from The Arabidopsis Information Resource repository^1^. Functional categorization of differentially represented proteins (DRPs) was obtained using Mercator pipeline^2^ for automated sequence annotation, selecting TAIR 10, SwissProt-UniProtKB plant proteins, KOG clusters and InterPro scan, with a cut-off value of 80. DRPs were assigned to Bevan functional classes (Bevan et al., 1998) using the classification from Mercator. Hierarchical clustering analysis of abundance ratio of DRPs at T0–T3 was performed using Genesis 1.8.1 platform (Sturn et al., 2002). Person’s correlation as distance and average linkage clustering were chosen as parameters. Protein interaction networks were obtained with STRING^3^ using the *A. thaliana* database.

## Results and Discussion

Based on the above-mentioned considerations regarding the recognition of independent C_2_H_4_-dependent and low temperature-modulated ripening mechanisms in kiwifruit (Asiche et al., 2018), the widest use of solely temperature-centered storage procedures in the corresponding industry to slow-down the ripening process and extend fruit life (Pranamornkith et al., 2012; Asiche et al., 2017), and the occurrence of previous proteomic studies already describing the effect of combined treatments using both low temperature and chemical additives on berries, which were finally removed from cold storage and allowed to ripe at 20°C (Minas et al., 2012, 2014, 2016; Tanou et al., 2015; Ainalidou et al., 2016), we focused our attention on kiwifruit whose postharvest management was limited to the application of a low temperature (4°C). This temperature value was chosen to avoid any risk of fruit freezing. Cold stored fruit samples were then taken at different times of postharvest treatment (T0–T3) and were subjected to analysis of pomological/compositional characteristics and proteometabolomic composition.

Results from the analysis of kiwifruit pomological/ compositional features at T0–T3 are reported in Table 1. These data demonstrated that fruit weight and firmness progressively decreased during cold storage. In particular, weight loss showed a reduction of 1.8, 3.8, and 5.7% after 30, 60, and 90 days of storage, respectively. Our results were in good agreement with previous observations on the same cultivar regarding the effect of cold conditions on fruit weight and firmness (Chiaramonti and Barboni, 2010; Lim et al., 2016; Asiche et al., 2017). On the other hand, we observed a progressive increase of soluble solid content (SSC) (Table 1), which reached a value 14.3 °Brix after 90 days. Also in this case, our results were in good agreement with previous investigations (Chiaramonti and Barboni, 2010; Mworia et al., 2012; Asiche et al., 2017), which demonstrated an increase of the SSC value during various regimes of cold storage. Finally, no significant differences were observed in Chroma, Hue angle and protein content values in the range T0–T3 (Table 1), confirming previous observations (Ghasemnezhad et al., 2013). Regarding C_2_H_4_ production in kiwifruit, data reported in Table 1 show that gas emission was appreciable during the whole period of fruit cold storage. In particular, C_2_H_4_ was detected even at T0–T1, and followed a production curve resembling that of other climacteric fruits, with low levels in the early storage (T1 and T2), followed by a sharp increase at T3 (climacteric peak). Also in this case, measured data were coherent with previous determinations on kiwifruit samples subjected to cold storage conditions (Chiaramonti and Barboni, 2010; Lim et al., 2016; Asiche et al., 2017).

**Table 1:** Pomological and qualitative traits of kiwifruits taken at postharvest stages T0, T1, T2, and T3 following cold storage at 4°C.

Time storage (days)	Weight loss (%)	Firmness (N)	SSC (°Brix)	Chroma	Hue angle	TP (mg/g FW)	Ethylene concentration (ppm)
0	–	71.2 ± 2.1^d^	7.4 ± 0.3^a^	50.8 ± 1.1^a^	109.2 ± 4.6^a^	0.59 ± 0.08^a^	0.02 ± 0.01^a^
30	1.8 ± 0.4^a^	45.5 ± 1.5^c^	10.9 ± 0.8^b^	51.2 ± 1.5^a^	110.7 ± 3.3^a^	0.53 ± 0.09^a^	0.07 ± 0.02^b^
60	3.8 ± 0.5^b^	32.5 ± 1.7^b^	12.5 ± 0.4^c^	50.3 ± 1.2^a^	112.8 ± 3.1^a^	0.58 ± 0.05^a^	0.09 ± 0.01^b^
90	5.7 ± 0.6^c^	22.3 ± 1.9^a^	14.3 ± 0.5^d^	50.7 ± 1.4^a^	115.3 ± 2.7^a^	0.52 ± 0.06^a^	0.39 ± 0.03^c^


*Reported are data on fruit weight loss (%), fruit firmness (N), total soluble solid content (SSC), Chroma, Hue angle, total protein content (TP) and ethylene emission during storage. Reported are mean values ± SD; mean values followed by the same letter do not differ significantly at P = 0.05 (Tukey’s Test).*

### Metabolomics

**FIGURE 1:**
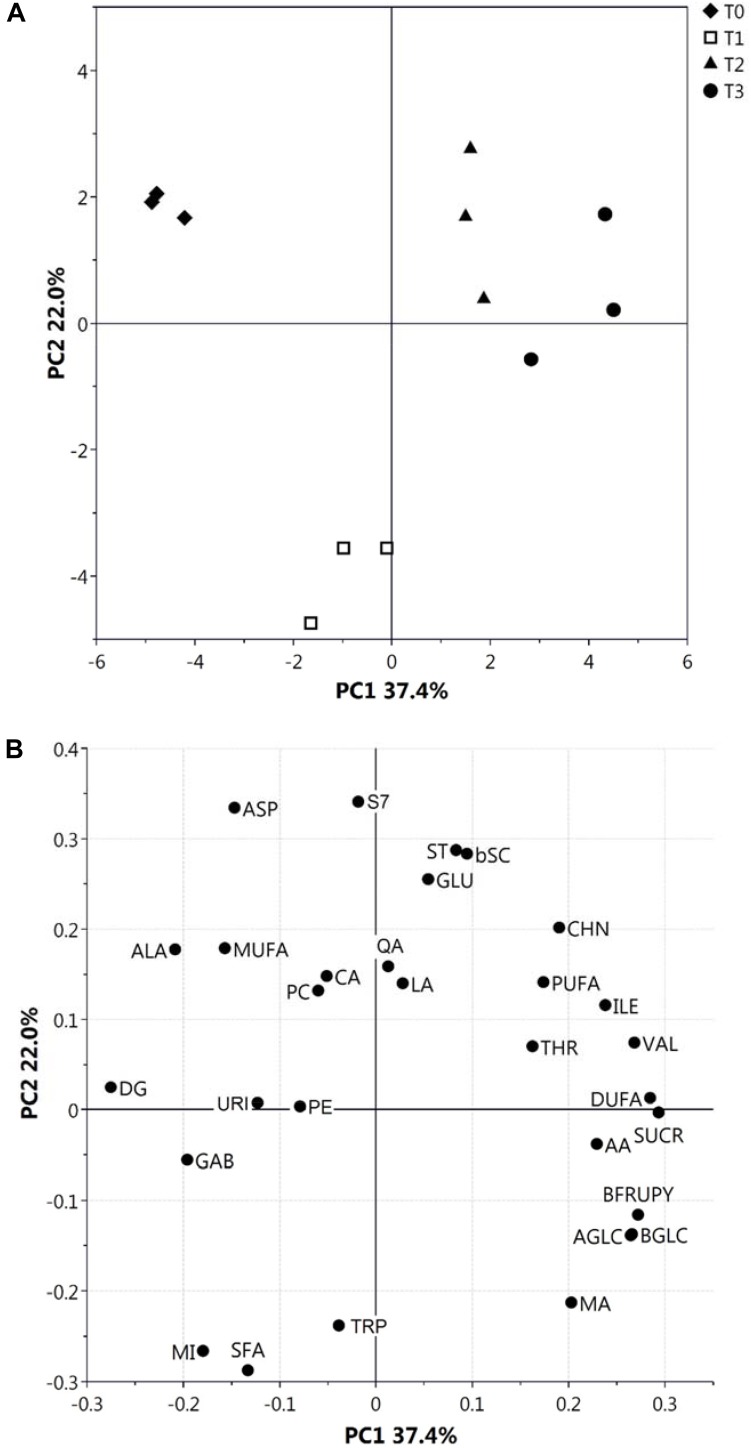
PCA score **(A)** and loading **(B)** plots of metabolite profiles (30 metabolites/metabolite classes) obtained from NMR data related to hydroalcoholic and organic extracts of kiwifruit at different postharvest stages (T0–T3). Results associated with malic acid (MA), citric acid (CA), ascorbic acid (AA), lactic acid (LA), quinic acid (QA), α- and β-glucose (AGLC and BGLC), sucrose (SUCR), β-fructopyranose (BFRUPY), Ala, Thr, Glu, Asp, Val, Ile, Trp and γ-aminobutyric acid (GAB), choline (CHN), uridine (URI), myo-inositol (MI), phosphatidylcholine (PC), phosphatidylethanolamine (PE), digalactosyl-diacylglycerol (DG), stigmast-7-en-3β-ol (S7), stigmasterol (ST), poly-unsaturated fatty acids (PUFA), di-unsaturated fatty acids (DUFA), mono-unsaturated fatty acids (MUFA), saturated fatty acids (SFA), and β-sitosterol plus campesterol (bSC) are shown.

We previously used NMR to evaluate metabolite concentration changes during kiwifruit physiological development (Salzano et al., 2018). The same approach was used here to quantify metabolites in hydroalcoholic (free amino acids, organic acids, sugars and others) and organic (fatty acids, phospholipids, sterols, and galactolipids) extracts at different postharvest stages. Almost all metabolites observed in the same extracts during fruit development (Salzano et al., 2018) were also present at T0–T3; unique exceptions were Gln, Asn, Phe, and shikimic acid, which were not detected in this study. Metabolite levels at T0–T3 stages are reported in Supplementary Figures S1, S2. Quantitative data were submitted to PCA (Figure 1), which provided a general view of metabolite changes associated with postharvest cold storage of kiwifruit. In particular, the first principal component accounted for 37.4% of total variability (Figure 1A) and it was strictly associated with postharvest, as shown by grouping of fruit samples according to the postharvest stage along PC1 axis. As observed by the plot of loadings, variables responsible for this trend were sugars (SUCR, AGLC, BGLC, and BFRUPY), AA, Thr, Ile, Val, and DUFA (see the legend to Figure 1 for abbreviations), which were present at a higher level during the final stages of postharvest (Supplementary Figures S1, S2); conversely, Ala and DG showed the highest level at T0. Data on SUCR, AGLC, BGLC, BFRUPY, and AA were coherent with previous determinations on total sugars and AA in postharvest kiwifruit stored at low temperatures (Chiaramonti and Barboni, 2010; Ghasemnezhad et al., 2013). Worth mentioning is the fact that T1 samples were characterized by distinct PC2 levels in the PCA score plot, due to a particular trend of few metabolites reaching the highest (SFA, MI, and Trp) or the lowest (Asp, Glu, and S7) level at T1. As expected, the trend showed by single metabolites (Supplementary Figures S1, S2) confirmed the results of PCA. In detail, AGLC, BGLC, and BFRUPY showed a relevant increase only during the first stage, being relatively constant in the later stages, whereas SUCR showed a constant increment during the whole postharvest period. The level of LA, QA, and CA remained unchanged in the T0–T3 range, whereas a slight increment of AA concentration was observed at T3 stage. Regarding the organic extracts, a constant increment and decrement of DUFA and DG was observed, respectively, whereas the other metabolites remained practically unchanged (Supplementary Figure S2).

**FIGURE 2:**
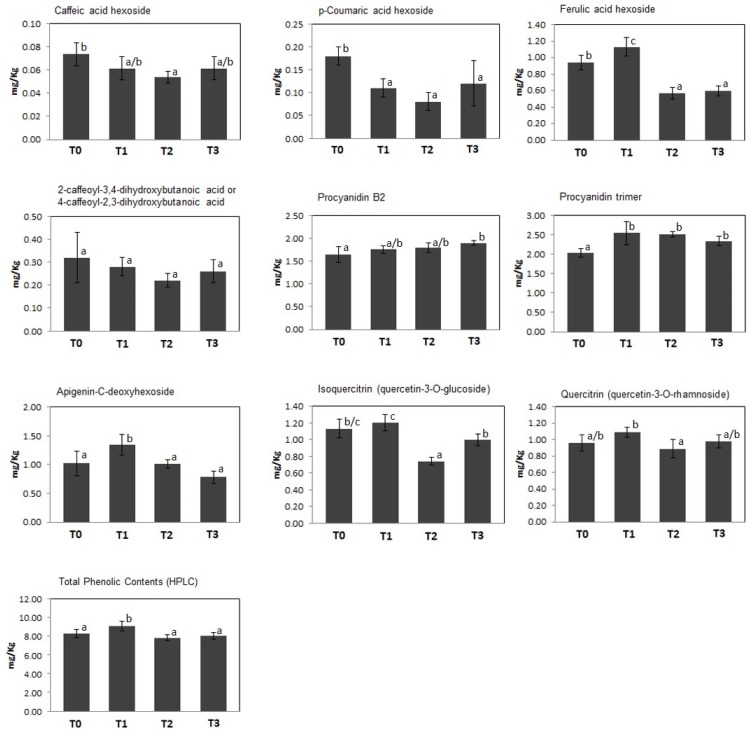
Concentration (mg/Kg FW) of individual and total polyphenols identified in kiwifruit extracts at different postharvest stages (T0–T3). Histograms represent the mean ± SD (*n* = 6; 3 biological replicates measured twice). Different letters on the bars indicate significant differences of polyphenol concentrations within the postharvest stage (Tukey’s test, *p* ≤ 0.05).

A quantitative measurement of polyphenolics present in kiwifruit at T0–T3 was achieved through their determination and characterization by HPLC-UV and ESI–ITMS^n^ analysis, respectively. Thus, 9 compounds were identified through the recognition of their diagnostic MS and MS^n^ signals; the classes of phenolic metabolites detected in this study perfectly matched those already described in kiwifruit undergoing physiological development (Salzano et al., 2018). In particular, 4 principal groups were detected, namely phenolic acids (caffeic acid hexoside, *p*-coumaric acid hexoside, ferulic acid hexoside, 2-caffeoyl-3,4-dihydroxybutanoic acid or 4-caffeoyl-2,3-dihydroxybutanoic acid), procyanidins (procyanidins B2 and trimer), flavones (apigenin-*C*-deoxyhexoside), and flavonols (quercetin-3-*O*-glucoside and quercetin-3-*O*-rhamnoside). Total and individual polyphenol amounts in kiwifruit pulp extracts at T0–T3 are shown in Figure 2. At T1, kiwifruits showed a slight increase in total phenol content (about 15%, from 8.29 ± 0.45 mg/kg of FW at T0 to 9.54 ± 0.34 mg/kg of FW at T1), which was followed by a slight decrease at T2 and T3 (7.87 ± 0.34 mg/kg of FW and 8.04 ± 0.35 mg/kg of FW, respectively). At the harvest stage (T0), procyanidins were the most predominant molecular class (3.68 ± 0.04 mg/kg of FW), accounting for about 44% of total phenolic content, followed by flavonols, whose total content was 2.09 ± 0.01 mg/kg of FW. Phenolic acids and flavones were found in minor amounts (1.51 ± 0.05 mg/kg of FW and 1.02 ± 0.21 mg/kg of FW, respectively) (Figure 2). This trend remained throughout the whole cold storage period (T1, T2, and T3). In agreement with data on total phenol content, above-mentioned polyphenolic classes showed a slight increase after 30 days of cold storage (phenolic acids about +4.6%, procyanidins about +17%, flavones about +32% and flavonols about +9.6%; increase from T0 to T1), which was followed by a certain decrease in the following time (phenolic acids about -34%, flavones about -42% and flavonols about -13,5%; decrease from T1 to T3). Unique exceptions were procyanidins, whose values remained almost constant.

In the whole, this study provided original information on quantitative levels of amino acids, sugars, organic acids, saturated/unsaturated fatty acids, phospholipids, sterols, galactolipids, phenolic acids, procyanidins, flavones, flavonols, and other metabolites in postharvest kiwifruit subjected to cold storage. These results were coherent with previous results on total reducing sugars and AA in postharvest fruit stored under similar experimental conditions (Chiaramonti and Barboni, 2010; Ghasemnezhad et al., 2013; Hu et al., 2016). Eventual discrepancies observed for AA and phenolic compounds may depend on the specific cultivar investigated and/or the non-specific assays authors used, with respect to our compound-oriented determination (Ghasemnezhad et al., 2013; Park et al., 2015b; Lim et al., 2016). In the whole, metabolite changes observed during kiwifruit storage at 4°C were in good agreement with molecular function, as related to fruit physiology (Richardson et al., 2011; Huang et al., 2013). Individual metabolites will be discussed below, together with proteins, corresponding metabolic pathways and related physiological processes.

### Proteomics

**FIGURE 3:**
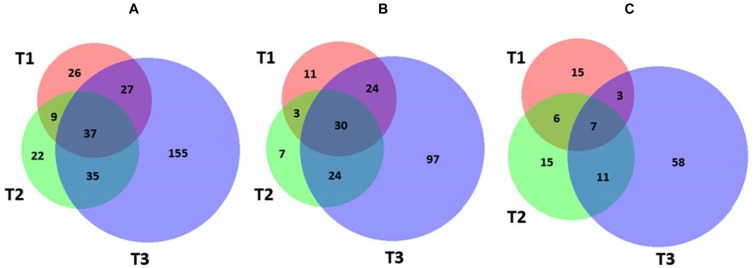
Venn diagram showing differentially represented spots among the different kiwifruit postharvest stages (T1, T2, and T3 in comparison with T0). Diagrams refer to all DRSs **(A)**, those over-represented **(B)**, and down-represented **(C)**, respectively.

In order to identify molecular effectors/metabolic pathways deregulated as result of kiwifruit cold storage, protein extracts from fruit samples taken at T0 (reference) and T1–T3 were comparatively evaluated by 2D-DIGE (Supplementary Table S1). Corresponding proteomic maps showed the presence of almost 4100 spots occurring within M*r* and p*I* ranges of 10–100 kDa and 3–10, respectively (Supplementary Figure S3). These spots were further filtered for abundance fold changes ≥1.5 or ≤-1.5 (T1–T3 vs. T0) and *p*-value ≤ 0.05 (Student’s paired *t*-test), ascertaining 311 differentially represented ones (DRSs) associated with cold storage (Supplementary Figure S3). Practically, most spots differentially represented at T1–T3 were already present at T0, suggesting that molecular processes modified in the course of kiwifruit cold storage were already active in the corresponding initial phase. Venn diagram showed unique and shared DRSs at the different postharvest stages (Figure 3). Hierarchical clustering of spot abundance ratios highlighted that most significant quantitative variations occurred at T3, which corresponded to apex in C_2_H_4_ emission (Supplementary Figure S4 and Table 1). After running of a preparative 2-DE, 2D-DIGE software modules allowed matching the corresponding image with that of the analytical counterpart. In 299 cases, MS analysis of the tryptic digest from DRSs led to protein identification (Supplementary Table S2). Since some DRSs showed the occurrence of two comigrating proteins within the same spot, results were further filtered as reported in the experimental section to exclude: (i) spots containing molecular species with no coherent quantitative levels in 2D-DIGE; (ii) comigrating proteins having a unique quantitative determination as deduced by 2D-DIGE (Supplementary Table S3). This conservative approach limited useful proteomic data to 235 spots, corresponding to 328 non-redundant sequence entries in the kiwifruit genome (Huang et al., 2013). As expected, although significant in number, differentially represented proteins ascertained during cold storage of harvest kiwifruit were lower in number than counterparts in fruit during physiological development (Salzano et al., 2018).

**FIGURE 4:**
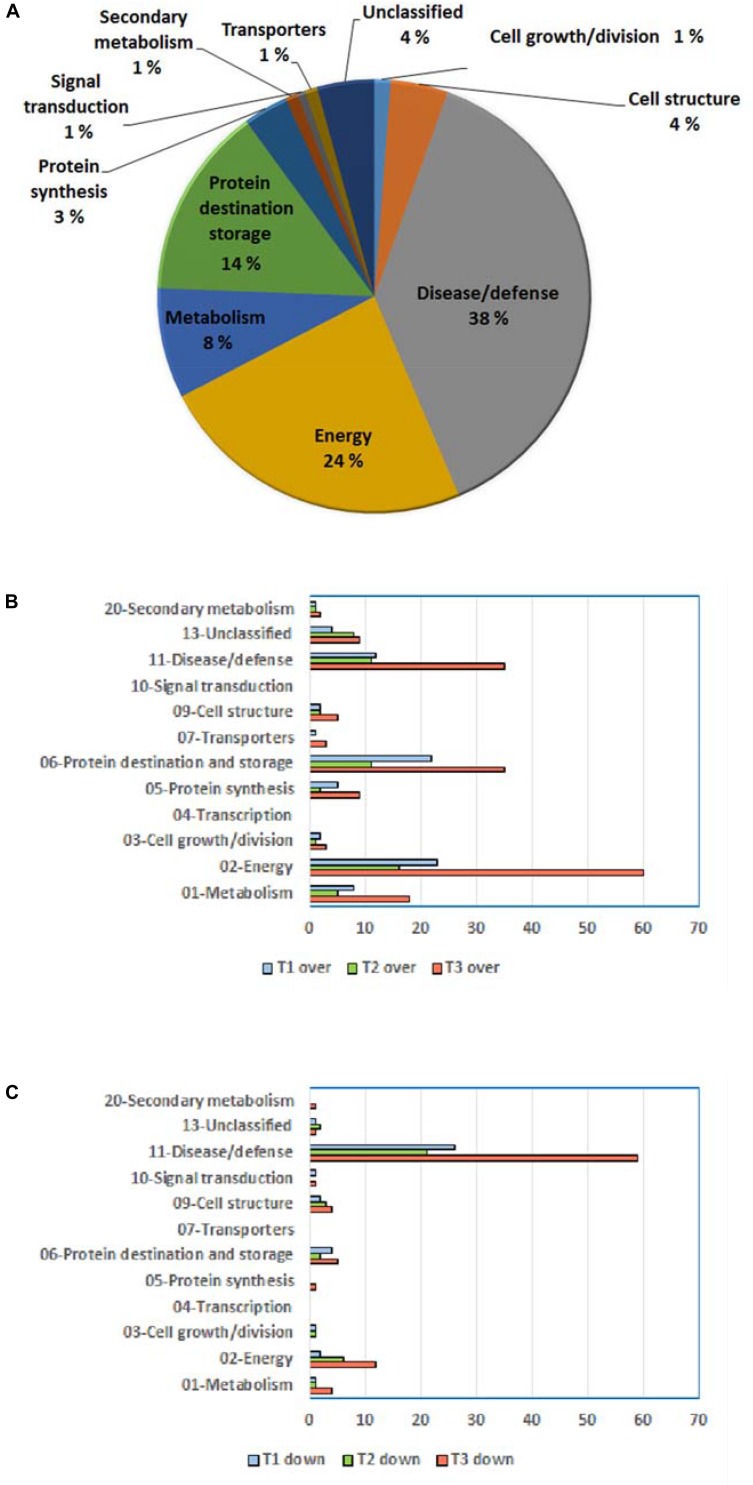
General information on differentially represented proteins (T1, T2, and T3 vs. T0). **(A)** Distribution of DRPs according to Bevan classification (Bevan et al., 1998). Distribution of functional categories of DRPs during different development stages for over-represented **(B)** and down-represented **(C)** proteins.

Resultant DRPs were indexed based on Bevan functional cataloging (Bevan et al., 1998) through an initial functional assignment obtained from Mercator software analysis. This analysis automatically attributed a function to all proteins, except for 17 that had not been assigned to any known ontology or function. According to their identity (and incidence > 5% in DRSs), DRPs were related to the Bevan functional category of: (i) disease/defense (38%); (ii) energy (24%); (iii) protein destination and storage (14%); (iv) metabolism (8%); (v) cell structure (4%), underlining the prominent molecular mechanisms modified during kiwifruit cold storage (Figure 4A). Distribution of functional groups of DRPs at T1–T3 is reported in Figure 4B,C. Among down-represented proteins, most relevant groups were disease/defense (T1, T2, and T3) and energy (T3). Over-represented counterparts were generally more abundant at T3 than at previous stages, most of them belonging to energy, protein destination and storage, disease/defense and metabolism categories. A heat-map picture originated from hierarchical clustering of quantity ratios of DRPs for each functional group during kiwifruit cold storage is shown in Supplementary Figure S5; it describes the dynamic expression profile of the various proteins among different storage stages. In subsequent paragraphs dedicated to the most significant protein functional categories, this figure is widely discussed together with corresponding DRPs and metabolites.

**FIGURE 5:**
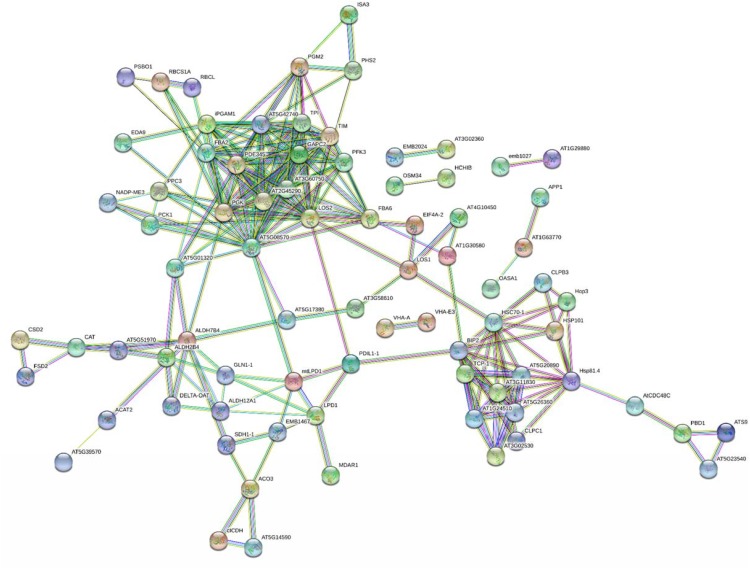
STRING analysis of differentially represented proteins in kiwifruit at T1, T2, and T3 with respect to T0. Only-high confidence interactions (0.7) are evidenced. Abbreviations are reported in Supplementary Table S4.

A bioinformatic prediction of protein–protein interactions among *A. thaliana* homologs of here-ascertained kiwifruit DRPs revealed a highly-ramified network bridging 98 sequence entries (Figure 5 and Supplementary Table S4), which corresponded to four main functional groups partially structured into three subnetworks. The assemblies of energy (39), protein destination and storage (19), stress/defense (15) and metabolism (12) comprised the highest number of entries. This finding underlined the occurrence of a functional assembly linking together various deregulated metabolic pathways and molecules involved in physiological response of kiwifruit to cold storage. An analysis of the heat-map representation (Supplementary Figure S5) of the interacting proteins within the main network showed a general common dynamic trend over time, mainly concentrated at T3. Overall, proteomic results pointed out that various energetic, metabolic and structural processes, together with plant defensive/stress-responsive mechanisms, are temporarily regulated during prolonged kiwifruit cold storage to elicit specific physiological mechanisms associated with this postharvest management.

#### Stress/Defense Proteins

Recently, we have demonstrated that kiwifruit physiological maturation is associated with a general increase of defensive proteins, which are synthesized to let the fruit adapt and resist to possible adverse abiotic/biotic stresses (Salzano et al., 2018). These processes are often related to augmented levels of ROS (Bachi et al., 2013) as well as of various antioxidant enzymes, which are induced in berry cells to neutralize the toxic activity of above-mentioned noxious compounds (Rinalducci et al., 2008). Cold storage of harvest kiwifruit induced an opposite quantitative trend for superoxide dismutase [Cu-Zn] (CSD2) and [Mn-Fe] (FSD2), glutathione *S*-transferase (GSTL3), putative glutathione *S*-transferase (GSTF7), abscisic stress ripening protein (AT1G70810), catalase 3 (CAT), quinone oxidoreductase wrbA, dehydrin 2 (COR47) and Cbs domain protein (CBSX3), suggesting that the prominent role of redox stress processes underpin by these proteins during fruit physiological maturation (Jimenez et al., 2002; Palma et al., 2011; Molassiotis et al., 2013) may be reduced in the postharvest cold storage phase. In fact, these components become progressively down-represented at T2 and T3 (Supplementary Table S3 and Supplementary Figures S5A,F). Most of the above-reported proteins are ROS-scavenging species participating in the direct response of plant cells to these xenobiotics and/or their derivatives (Carey et al., 2007; Kim et al., 2012; Bachi et al., 2013; Liu et al., 2017), enzymes catalyzing the conjugation of these toxic molecules to glutathione (GSH) (Bachi et al., 2013), or effectors regulating the activation of the thioredoxin system controlling H_2_O_2_ levels (Yoo et al., 2011). On the other hand, a coherent opposite behavior with respect to kiwifruit physiological maturation was also observed for monodehydroascorbate reductase (MDAR1), which showed an augmented representation at T3 (Supplementary Table S3 and Supplementary Figure S5A). Since MDAR1 catalyzes the recycling conversion of monodehydroascorbate into AA (Noctor and Foyer, 1998), its increased levels well correlated with the rise of AA concentration measured during cold storage of harvest kiwifruit (Supplementary Figure S1). In this context, worth mentioning is the fact that AA, together with GSH, is an essential component of a dedicated molecular machinery detoxifying H_2_O_2_ and other peroxides (Noctor and Foyer, 1998), and has been reported to affect Met/C_2_H_4_ metabolism as well (see below) (Fercha et al., 2013). A similar quantitative profile was also verified for over-representation of cytosolic aldehyde dehydrogenase ALDH7B4 (AT1G54100) (Supplementary Table S3 and Supplementary Figure S5G), which is induced in plants as result of various abiotic stress conditions (Stiti et al., 2011). Measured level of specific proteins mentioned above was in good agreement with that observed in kiwifruit exposed to cold storage under different experimental conditions (Minas et al., 2016), in other climacteric fruits during ripening or cold storage (Nilo et al., 2010; Zhang et al., 2011; Giraldo et al., 2012; Zheng et al., 2013; Li et al., 2015; Du et al., 2016; Wu et al., 2016; Tanou et al., 2017) and in plants exposed to cold stress (Janmohammadi et al., 2015; Die et al., 2016, 2017). Some of the above-mentioned antioxidant proteins occurred in the interaction network shown in Figure 5. In the whole, our results suggest that the observed reduced representation of various antioxidant proteins in kiwifruit during cold storage may be associated with compensative regulation mechanisms paralleling the concomitant increased concentration therein of AA and constant representation of polyphenolics (Figure 1, 2), in a condition where redox stress does not seem having a prominent function or, more probably, is balanced by these antioxidant metabolites.

A more variable quantitative trend was observed for proteins involved in defense response to biotic stresses. In fact, while α-toxin, chitinase (HCHIB) and NtPRp27-like protein showed reduced levels at T1–T3, lectin exhibited an opposite condition, and abundant kiwellin, thaumatin-like protein isoforms (OSM34) and major latex-like protein (Bet VI class) presented a mixed profile, also associated with a decreased representation of corresponding proteolytic fragments (Supplementary Table S3 and Supplementary Figure S5A). By eliciting specific activities on the functional integrity of the cell wall (Mauch and Staehelin, 1989; Martínez-Esteso et al., 2009), membrane bilayer (Martínez-Esteso et al., 2009; Draffehn et al., 2013; Offermann et al., 2015) and the ribosomal machinery of exogenous hosts (Hartley and Lord, 2004), most of these proteins protect the fruit from microbial/fungal pathogens, and thus have been grouped as pathogenesis-related proteins (van Loon et al., 2006). These components showed similar quantitative trends in apple, banana, mango, and peach during physiological maturation (Zhang et al., 2011; Andrade et al., 2012; Zheng et al., 2013; Du et al., 2016) or in the same fruits and kiwifruit following cold storage under distinct postharvest conditions (Nilo et al., 2010; Giraldo et al., 2012; Li et al., 2015; Minas et al., 2016). These proteins can induce strong allergic reactions in humans and thus were identified among the most effective allergens in kiwifruit (Supplementary Table S5) (Bulley, 2016). Our findings suggest that cold storage of kiwifruit may influence quantitative representation of specific allergens, with important consequences at food consumption level. Thus, they have to be further considered in the development of dedicated studies on postharvest management of kiwifruit.

#### Protein Synthesis, Destination and Storage

The list of proteins involved in abiotic/biotic stress responses showing a differential representation in harvest kiwifruit after cold storage also included various chaperones, heat shock proteins (HSPs) and protein disulfide isomerases (PDIs) that, contributing to a proper polypeptide folding and disulfide pairing, were already described as being involved in plant defense against different environmental challenges (Supplementary Table S3 and Supplementary Figures S5A,B) (Timperio et al., 2008). Whereas protein disulfide isomerase-like (PDIL1-1) and stress-induced phosphoprotein (Hop3) showed reduced levels at T1 and T2, a number of other HSPs and chaperones were over-represented as result of kiwifruit cold storage. In particular, isoforms of HSP 70 kDa protein C (BIP2), HSP 90-2 (HSP81.4), 70 kDa HSP (HSC70-1), chaperone ClpB (CLPB3 and HSP101), and T-complex protein 1 subunits α (TCP-1), β (AT5G20890), γ (AT5G26360), ε (AT1G24510), η (AT3G11830), and ζ (AT3G02530) always showed augmented levels at T1, T2, and/or T3, whereas chaperone ClpB1 (CLPC1) was over-represented for the whole period of investigation. Some of the proteins reported above have already been referred as highly represented in banana, papaya and peach exposed to cold-based storage conditions (Huerta-Ocampo et al., 2012; Li et al., 2015; Wu et al., 2016) or in different plants following various abiotic stresses, including the cold one (Somer et al., 2002; Renaut et al., 2006; Timperio et al., 2008; Janmohammadi et al., 2015; Die et al., 2016, 2017; Muthusamy et al., 2016). They are known to assist polypeptide synthesis and macromolecular structures assembly, and to prevent corresponding misfolding in environmental conditions hampering their function. Above-mentioned species were present as a subnetwork in Figure 5, and were linked to other components involved in protein synthesis and protein destination/storage.

To maintain proper cell functioning in an environmental stress condition (cold storage) eventually associated with formation of misfolded/non-soluble polypeptide species, various molecular machineries were also regulated in harvest kiwifruit at T1–T3 to ultimately lead to a condition where optimal protein turnover was ensured, but total protein levels remained unaltered (Table 1). In the case of kiwifruit cold storage, these mechanisms seem to include the induction of various proteolytic enzymes, i.e., putative aminopeptidases (AT1G63770), subtilisin-like proteases (ARA12 and AT5G67090), acylamino-acid-releasing enzyme (AARE) and Xaa-pro aminopeptidase (APP1), and of components of the ubiquitin/26S proteasome machinery, namely 26S proteasome regulatory subunit (AT5G23540) and non-ATPase regulatory subunit (ATS9), which were over-represented at T1–T3 (Supplementary Table S3 and Supplementary Figure S5B). Concomitant repression of components of the ubiquitin-independent 20S proteasome system was also observed, namely subunit β isoforms (PBD1) and ubiquitin-conjugating enzyme MMZ3. In the latter context, worth mentioning is the fact that the 20S proteasome system, through the representation of its components, has already been reported to control the degradation efficiency of the ubiquitin/26S proteasome machinery in kiwifruit and grape (Renaut et al., 2006; Martínez-Esteso et al., 2011; Salzano et al., 2018). Thus, our results suggest that a reduced synthesis of members of the ubiquitin-independent 20S proteasome system, together with induction of components of the ubiquitin/26S proteasome system, may ultimately lead to a promoted activity of the latter in degrading misfolded/non-soluble polypeptide species. The probable occurrence of compensative mechanisms promoting protein synthesis under stressful conditions to maintain total protein levels unaltered fitted with the observation of increased levels of elongation factor isoforms LOS1 (LOS1), eukaryotic initiation factor 4A (EIF4A-1), ATP-binding cassette (ABCF1), Gly-tRNA ligase isoforms (AT1G29880), and Arg-tRNA ligase (emb1027) during kiwifruit cold storage (Supplementary Table S3 and Supplementary Figure S5C). The first protein was already identified as an inducible protein in kiwifruit experiencing C_2_H_4_ and chilling treatments (Minas et al., 2016). Protein homologs of the above-cited components showed similar quantitative trends in other plants exposed to cold (Renaut et al., 2006; Janmohammadi et al., 2015).

Finally, cysteine proteases cathepsin S (RD21B) and cathepsin B-like (RD21A), also known as actinidin and actinidin Act2a, respectively, showed reduced levels at T1–T3 (Supplementary Table S3 and Supplementary Figure S5B). Actinidins are among the most abundant proteins in physiologically ripe kiwifruit and banana, and were recognized as major allergens therein (Supplementary Table S5) (Bulley, 2016; Du et al., 2016). Their quantitative variation during cold storage again suggests that postharvest management of kiwifruit can influence quantitative representation of specific allergens, with important consequences on food allergic properties.

#### Cell Wall Remodeling Enzymes and Structural Proteins

Above-reported pomological data shows that cold storage of kiwifruit was associated with a reduction of its firmness (Table 1), confirming previous studies (Schröder and Atkinson, 2006; Chiaramonti and Barboni, 2010; Lim et al., 2016; Asiche et al., 2017). In agreement with these observations, we ascertained a differential representation of cell wall remodeling enzymes and structural proteins in the corresponding time range, complementing previous biochemical evidences (Martínez-Esteso et al., 2011). In particular, cell wall structural components, i.e., arabinogalactan protein (AT5G11680) and pro-resilin (AT5G39570), and cytoskeletal proteins, namely actin 1 (ACT7), annexin (ANNAT4) and tubulin beta-2 chain (TUB5), showed reduced levels at T2–T3 (Supplementary Table S3 and Supplementary Figures S5D,M). Arabinogalactan protein is a hydroxyproline-rich glycoprotein, heavily modified by arabinose/galactose-rich polysaccharide chains and glycosylphosphatidylinositol anchors, which is cross-linked to pectin and pectin-arabinoxylan to ensure cell wall rigidity (Showalter and Basu, 2016). Above-reported findings were paralleled by over-representation of enzymes regulating cell wall pectin and hemicellulose degradation, namely pectinesterase-2 (PME51), pectinesterase inhibitor (PMEI2), β-xylosidase 4 (XYL4), polygalacturonase-inhibitor protein (PGIP1), putative uncharacterized proteins P0046B10.2-1 (AT3G08030 and AT5G11420) and anthranilate phosphoribosyltransferase (AT1G70570), which in some cases showed increased levels even at T1 or were characterized for their over-representation in T2–T3 range (Supplementary Table S3 and Supplementary Figures S5D,F,M). The activity of AT1G70570 has been reported to be regulated by C_2_H_4_ (Li et al., 2012). Most of these enzymes have been described to modulate the degree of methylesterification/acetylation and/or (consequent) polymerization of pectin homogalacturonans and hemicellulose xyloglucans (Sénéchal et al., 2014; Zúñiga-Sánchez et al., 2014; Genero et al., 2016). Cell wall-associated AT3G08030 contains a carbohydrate-binding domain and interacts with cell wall polysaccharides (Vázquez-Lobo et al., 2012). Our proteomic observation was in good agreement with previous studies on cell wall remodeling enzymes and structural proteins in cold exposed kiwifruit (Minas et al., 2016; Asiche et al., 2018) and in other climacteric fruits during ripening or subjected to a similar postharvest thermal management (Nilo et al., 2010; Zhang et al., 2011, 2012; Giraldo et al., 2012; Huerta-Ocampo et al., 2012; Du et al., 2016). All these evidences confirmed the occurrence of concomitant, distinct molecular mechanisms regulating cell wall disassembly and fruit softening in the fruit (Bennett and Labavitch, 2008).

#### Central Carbon and Energy Metabolism

Kiwifruit generally accumulates large amounts of starch during development (Richardson et al., 2011), which is then degraded in the postharvest phase (also during cold storage) (Richardson et al., 2011; Park et al., 2015b; Hu et al., 2016). In this context, progressively increasing SSC values ascertained at T1–T3 (Table 1) and corresponding sugar (SUCR, AGLC, BGLC, and BFRUPY) levels (Supplementary Figure S1) were coherent with the over-representation of enzymes involved in starch degradation and sucrose metabolism, namely glycogen debranching enzyme (ISA3), α-glucan phosphorylase (PHS2), phosphoglucomutase isoforms (PGM2) and phosphofructokinase (PFK3) (Supplementary Table S3 and Supplementary Figures S5F,G), thus providing a rationale to measured metabolite amounts. Above-cited proteomic changes were paralleled by increased levels of enzymes involved in glycolysis, i.e., glucose-6-phosphate isomerase (AT5G42740), fructose-bisphosphate aldolase isoforms 2 and 3 (PDE345, FBA6, and FBA2), triosephosphate isomerase (TPI), phosphoglycerate kinase isoforms (PGK), phosphoglycerate mutase isoforms (iPGAM1), enolase isoforms (LOS2) and pyruvate kinase (AT5G08570), and in the citric acid cycle, i.e., dihydrolipoyl dehydrogenase (mtLPD1), aconitate hydratase 2 (ACO3), isocitrate dehydrogenase [NADP] isoforms (CICDH and AT5G14590) and succinate dehydrogenase subunit A (SDH1-1), which were mostly constant at T1–T2 and then over-represented at T3. This ensured active metabolic pathways in harvest kiwifruit providing energy, cofactors and building blocks for fruit survival also during cold storage. Two GTP-binding proteins regulating glycolytic/TCA cycle pathways by a direct interaction with above-mentioned enzymes as well as chloroplast development, membrane fission and sensitivity to hormones were also over-represented in the same time range, namely GTPase obg (AT1G30580) and dynamin (DL1E) (Colaneri and Jones, 2014) (Supplementary Figures S5E,M). Regulation of glycolysis and the Krebs cycle pathways in kiwifruit was also ensured through down-representation of triosephosphate isomerase (TIM), glyceraldehyde-3-phosphate dehydrogenase (GAPC2) and dihydrolipoyl dehydrogenase (LPD1) (showing decreased levels at T1 and T3, respectively) (Supplementary Figures S5F,G).

Evidences for the activation of alcoholic fermentation during kiwifruit cold storage derived from ascertained over-representation of corresponding enzymes, i.e., Zn-containing alcohol dehydrogenase (AT4G13010), oxalyl-CoA decarboxylase (AT5G17380), and pyruvate decarboxylase 2 (AT5G01320). In particular, increased levels of AT5G01320 were coherent with promoted conversion of pyruvate from glycolysis into toxic acetaldehyde, which then was converted into ethanol by the detoxifying action of augmented AT4G13010, also enabling production of NADH, or transformed into acetate by increased levels of aldehyde dehydrogenase ALDH2B4 (AT3G48000) (Wei et al., 2009; Stiti et al., 2011) (Supplementary Table S3 and Supplementary Figures S5F,G). AT5G01320 and AT4G13010 have already been reported being induced in apple following C_2_H_4_ treatment (Yang et al., 2016). Conversely, a down-representation of enzymes involved in photosynthesis was observed, namely ribulose bisphosphate carboxylase large chain (RBCL), ribulose bisphosphate carboxylase small chain (RBCS1A), and oxygen-evolving enhancer protein 1 (PSBO1); a similar trend was also observed for plastid-lipid-associated protein (AT4G22240) and chloroplastic outer envelope pore protein 24 (AT1G45170) (Supplementary Figures S5D,G,M). These proteins are directly involved or stabilize plastid machineries essential in providing the energetic supply of the fruit and in maintaining its endogenous O_2_ balance. Our results suggest that photosynthesis is not a preferential energetic pathway in harvest kiwifruit during cold storage, confirming previous observations based on gene expression data on fruit postharvest at room temperature (Richardson et al., 2011). Nevertheless, energy supply in fruit seemed guaranteed by dedicated compensative mechanisms, as evidenced by increased levels measured for V-type ATP synthase alpha chain (VHA-A), V-type proton ATPase subunit E (VHA-E3) and NADH-ubiquinone oxidoreductase isoforms (EMB1467) isoforms in the T1–T3 range (Supplementary Table S3 and Supplementary Figures S5G,L). Enzymes involved in the C4 cycle, namely malic enzyme (NADP-ME3) and phosphoenolpyruvate carboxylase (PPC3), also showed over-representation at T2 and T3, whereas proteins assisting pentose-phosphate metabolism displayed a mixed quantitative trend overtime, i.e., 6-phosphogluconate dehydrogenase (AT3G02360), 6-phosphogluconolactonase (EMB2024), and transketolase isoforms (AT3G60750 and AT2G45290).

In the whole, the quantitative behavior of specific proteins involved in glycolysis, the Krebs cycle, alcoholic fermentation, energy production and additional carbon metabolism pathways in kiwifruit well paralleled that observed in the same fruit exposed to low temperatures but in different experimental setup (Minas et al., 2016; Asiche et al., 2018), in other fruits during ripening (Borsani et al., 2009; Andrade et al., 2012; Huerta-Ocampo et al., 2012; Nogueira et al., 2012; Yun et al., 2013; Zheng et al., 2013; Du et al., 2016; Xiao et al., 2018) or cold-based postharvest management (Nilo et al., 2010; Li et al., 2015; Wu et al., 2016; Tanou et al., 2017; Wang et al., 2017), or in other plants experiencing cold stress conditions (Janmohammadi et al., 2015; Die et al., 2016, 2017), suggesting the existence of common regulation mechanisms of these metabolic pathways in above-mentioned organisms. Most of the proteins reported above constitute two functional subnetworks linked to each other and to additional ones related to stress response and protein destination/storage (Figure 5).

#### Other Metabolic Enzymes

Significant differences in amino acid content were observed during kiwifruit cold storage. In fact, Val, Ile, Thr, Ala, Trp, Asp, Glu, and GAB showed variable quantitative levels overtime, which were also different with respect to counterparts ascertained during fruit physiological development (Supplementary Figure S1). Proteomic results at T1–T3 were frequently indicative of the modulation of the corresponding anabolic/catabolic pathways (Supplementary Table S3 and Supplementary Figures S5F–H). For example, Val and Ile increased during kiwifruit cold storage in parallel to over-representation of Val/Leu/Ile biosynthetic enzymes ketol-acid reductoisomerase (AT3G58610) and 3-isopropylmalate dehydratase large subunit (AT2G05710). Similarly, increased concentration of Trp at T1 was in good agreement with ascertained augmented levels of the biosynthetic enzyme anthranilate phosphoribosyltransferase (AT1G70570) at that time and, at T3, of polyphenol oxidase (NdhS) degrading oxidized protein adducts, which was also possibly explicative for slight augmented concentration of specific polyphenolics at the final stage of kiwifruit cold storage. On the other hand, accumulation of Thr overtime was associated with the decreased representation of L-threonine 3-dehydrogenase (AT5G51970), which is involved in irreversible degradation of this amino acid. More complex was the tentative explanation of Asp, Ala, Glu, and GABA levels in the course of kiwifruit cold storage (Supplementary Figure S1), based on multiple (overlapping) metabolic pathways in which these amino acids are involved (Ainalidou et al., 2016), and the limited information on corresponding enzyme representation trends (Supplementary Table S3 and Supplementary Figure S5F). Finally, the increased levels at T3 of cytosolic cysteine synthase (OASA1), which catalyzes Cys biosynthesis, were associated with its partial involvement in the removal of cyanide formed as result of 1-aminocyclopropane-1-carboxylate-oxidase (ACO)-dependent production of C_2_H_4_, in agreement with what observed in kiwifruit and other fruits subjected to various postharvest managements (Jost et al., 2000; Du et al., 2016). The quantitative levels of the latter enzyme provided a rationale to the amounts of C_2_H_4_ measured in the range T0–T3 (Table 1), underlying the essential role of ACO4 in C_2_H_4_ biosynthesis (Atkinson et al., 2011), and the activation of this enzyme in the experimental conditions used in this study for kiwifruit cold storage. In this context, worth mentioning is the fact that our experiments were performed in the absence of C_2_H_4_ receptors inhibitors (i.e., 1-methylcyclopropene), thus missing the possibility to discriminate between distinct C_2_H_4_-dependent and low temperature-dependent ripening mechanisms (Asiche et al., 2018). Over-representation of above-mentioned proteins and C_2_H_4_ well paralleled with a concomitant increase of AA concentration in kiwifruit during cold storage (Supplementary Figure S3), further supporting the important relation between AA, ROS, and C_2_H_4_-regulated enzymes during fruit postharvest (Lum et al., 2016). Some of the above-cited results paralleled what observed in the same fruit exposed to low temperatures but in different experimental setup (Asiche et al., 2018), in other fruits during ripening (Nogueira et al., 2012; Zhang et al., 2012; Zheng et al., 2013; Du et al., 2016) or cold-based postharvest management (Nilo et al., 2010; Tanou et al., 2017), or in plants subjected to cold stress (Die et al., 2016); they were suggestive of specific metabolic steps where regulation of amino acid or secondary metabolite anabolism/catabolism is exerted.

On the other hand, different enzymes involved in purine/pyrimidine catabolism were over-represented in T1–T3 range, namely uricase (urate oxidase) (AT2G26230) and dihydropyrimidase (PYD2) (Supplementary Figure S5F), in agreement with analogous reports on other fruits during postharvest management or in plants subjected to cold stress. The first enzyme is involved in uric acid oxidation, thus removing this sparingly soluble plant metabolite, while the second one is involved in uridine degradation, providing a rationale to the decreased levels of this pyrimidine measured overtime (Supplementary Figure S3).

Regarding kiwifruit proteins involved in secondary metabolite anabolism/catabolism, proteomic data showed an over-representation at T1–T2 of cinnamyl alcohol dehydrogenase 4 (ATCAD4), as already observed during ripening of banana (Du et al., 2016), whereas 3-ketoacyl-CoA thiolase (ACAT2) and rubber elongation factor (REF) showed increased levels even at earlier stages (Supplementary Table S3 and Supplementary Figures S5F,H,M). ATCAD4 catalyzes NADPH-dependent reduction of caffeyl aldehyde to its respective alcohol in corresponding phenylpropanoid biosynthetic pathway; its abundance in kiwifruit during cold storage was related to the decrease of caffeic acid hexoside concentration ascertained therein (Figure 2). REF is a protein assisting poly-isoprene polymerization in the monolayer membrane of rubber, which also presents allergic properties (Berthelot et al., 2012) whereas ACAT2 is involved in isoprenoid/terpenoid pathway that supplies precursors for the biosynthesis of carotenoids, dolichols, and sterols. Over-representation of ACAT2 during kiwifruit cold storage was tentatively associated with increased or, at least, constant concentration of S7, ST, and bSC, respectively (Supplementary Figure S2), although a rationale justifying the specific quantitative levels overtime of these sterols was not deduced.

## Conclusion

By using combined NMR/LC-UV/ESI-IT-MS^n^ and 2D-DIGE/nLC-ESI-LIT-MS/MS procedures, this study provides a global picture of the metabolite and protein quantitative changes occurring during kiwifruit cold storage, under conditions not using additional treatment with specific chemicals. About 42 metabolites were evaluated, showing in some cases concentration trends similar to that previously reported. In parallel, protein representation results allowed revealing that components related to disease/defense, protein destination and storage, metabolism, energy and cell structure functions were highly affected at specific moments of kiwifruit postharvest management. A number of these components occurred in a predicted functional interaction network that, based on ascertained results, appears to orchestrate protein representation overtime to modulate essential reactions underlying kiwifruit during its postharvest life and/or its adaptation to cold conditions. Most protein quantitative variations occurred in correspondence of the apex in C_2_H_4_ emission, underlining the prominent role of this phytohormone in fruit physiology. Protein representation trends also provided an explanation to some pomological characteristic and metabolite concentration variations, integrating previous studies on this (Minas et al., 2012, 2014, 2016; Tanou et al., 2015; Asiche et al., 2018) and other fruits (Pedreschi et al., 2008, 2009; Borsani et al., 2009; Lara et al., 2009; Costa et al., 2010; Nilo et al., 2010; Zhang et al., 2010, 2011, 2012; Andrade et al., 2012; Giraldo et al., 2012; Huerta-Ocampo et al., 2012; Nogueira et al., 2012; Yun et al., 2013; Zheng et al., 2013; Cai et al., 2014; Jiang et al., 2014; Lauxmann et al., 2014; Li et al., 2015; Du et al., 2016; Liu et al., 2016; Sun et al., 2016; Wu et al., 2016; Tanou et al., 2017; Wang et al., 2017; Xi et al., 2017; Xiao et al., 2018). Since a number of metabolites and proteins for which a metabolic/functional linkage was hypothesized in physiological ripening of kiwifruit (Salzano et al., 2018) showed a concomitant opposite quantitative behavior with respect to that reported in this study, the results presented here reinforce our previous hypotheses on their mutual functional association. By providing an original proteometabolomic description of harvest kiwifruit during cold storage under conventional postharvest management, this investigation provides a picture of fruit physiology in a condition that is generally adopted from kiwifruit industry, integrates previous important studies on this berry based on different postharvest management procedures (Minas et al., 2012, 2014, 2016; Tanou et al., 2015), and add additional insights on the evaluation of metabolic pathways and molecular effectors in harvest fruits from other species according to a holistic perspective (Hertog et al., 2011; Pedreschi, 2017).

## Author Contributions

AMS, GR, VC, MP, APS, LM, and AS designed the experiments, analyzed the data, and wrote the manuscript. AMS, GR, APS, DC, MV, GN, and MSP performed the experiments. VC, MP, NZ, LM, and AS commented on the manuscript. All authors read and approved the manuscript.

## Conflict of Interest Statement

The authors declare that the research was conducted in the absence of any commercial or financial relationships that could be construed as a potential conflict of interest.

## Footnotes

1 www.arabidopsis.org

2 http://mapman.gabipd.org/web/guest/app/mercator

3 http://string-db.org

## Abbreviations

AA: ascorbic acid
C_2_H_4_: ethylene
FA: formic acid
MS: mass spectrometry
ROS: reactive oxygen species
