# Supplementary material for: Unveiling Kiwifruit Metabolite and Protein Changes in the Course of Postharvest Cold Storage
Source: Front Plant Sci. 2019 Feb 4;10:71. doi: 10.3389/fpls.2019.00071 (PMC6369206; doi:10.3389/fpls.2019.00071)
Supplement: Supplementary file 2 [file Data_Sheet_1.pdf]

UNVEILING KIWIFRUIT METABOLITE AND PROTEIN CHANGES IN THE COURSE OF POSTHARVEST COLD STORAGE

Anna Maria Salzano *[1], Giovanni Renzone *[1], Anatoly P. Sobolev *[2], Virginia Carbone *[3], Milena Petriccione *[4], Donatella Capitani [2], Monica Vitale [1,5], Gianfranco Novi [1], Nicola Zambrano [5,6], Maria Silvia Pasquariello [4], Luisa Mannina [2,7] and Andrea Scaloni [1]

[1] Proteomics & Mass Spectrometry Laboratory, ISPAAM, National Research Council, 80147 Naples, Italy

[2] Magnetic Resonance Laboratory "Annalaura Segre", Institute of Chemical Methodologies, National Research Council, 00015 Monterotondo (RM), Italy

[3] Institute of Food Sciences, National Research Council, 83100 Avellino, Italy

[4] Centro di Ricerca per Olivicoltura, Frutticoltura e Agrumicoltura, Consiglio per la Ricerca in Agricoltura e l'Analisi dell'Economia Agraria, 81100 Caserta, Italy

[5] Dipartimento di Medicina Molecolare e Biotecnologie Mediche, Università degli Studi di Napoli "Federico II", 80131 Naples, Italy

[6] CEINGE Biotecnologie Avanzate, 80145 Naples, Italy

[7] Dipartimento di Chimica e Tecnologie del Farmaco, Sapienza Università di Roma, 00185 Rome, Italy

SUPPLEMENTARY MATERIAL

Materials and Methods

Chemicals

HPLC and LCMS-grade solvents were from Merck (Darmstadt, Germany) or Carlo Erba (Rodano, Milan, Italy). Gallic acid, caffeic acid, ferulic acid, cumaric acid and quercitrin (quercetin-3-O-rhamnoside) were purchased from Sigma Chemical Company (St. Louis, USA). Procyanidin B2 and isoquercitrin (quercetin-3-O-glucoside) were obtained from Extrasynthese (Genay, France) and Fluka (Buchs SG, Switzerlandand), respectively. HPLC grade water (18.2 MΩ) was prepared with a Millipore Milli-Q purification system (Millipore, Bedford, MA, USA).

Analysis of kiwifruit pomological and qualitative traits

Fruit weight was measured with an electronic balance having a 0.01 g-sensitivity, and used to calculate weight loss (%). Flesh firmness was measured at two opposite sides of fruits using a TR digital penetrometer (Turoni, Forlì, Italy) bearing a 8 mm-diameter probe; data are expressed in Newton (N). Outer pericarp colour was measured using a CR5M colorimeter (Minolta Camera, Japan), which allowed determining chromaticity L* (lightness), a* (green to red) and b* (blue to yellow) values at the opposite side of 30 kiwifruits for each postharvest stage. Hue angle and Chroma values were calculated from chromaticity a* and b* data (McGuire, 1992). Measurements were performed on 3 biological fruit replicates, each one consisting of 20 kiwifruits. Total soluble solid content (SSC, °Brix) was determined in the flesh juice of kiwifruits using a DBR35 digital refractometer (Sinergica Soluzioni, Pescara, Italy); measurements were performed on three technical replicates. Total proteins were extracted using a modified version of the phenol/SDS-based method (Wang et al., 2003; Salzano et al., 2018); protein content in fruits was measured with the Bio-Rad (Heracles, CA, USA) protein assay kit, based on the Bradford quantification method (Bradford, 1976), using bovine serum albumin as standard. Measurements were carried out in triplicate on 3 biological replicates, each one consisting of 10 kiwifruits.

Measurements of C_2H_4 production were performed on triplicates of three fruits that were placed into different airtight jars (2 l vol) for 30 min. A 1 ml headspace gas sample was withdrawn and injected in the splitless mode, using He as carrier gas (1 ml/min). Gas chromatography analysis was carried out using a Perkin-Elmer Autosystem XL instrument equipped with dual flame ionization detector (FID) system and a Porapak Q column (Waltham, USA). The oven temperature using isothermal program was set at 50 °C, for 15 min. Injector and detector temperature values were set at 280 °C.

Statistical analysis of pomological and qualitative data (one-way analysis of variance and Tukey's test for multiple mean comparisons) was performed using SPSS Software Package, version 20.0 (SPSS Inc., Chicago, IL, USA).

Extraction of kiwifruit metabolites and subsequent NMR analysis

Kiwifruit outer pericarp samples (about 2 g) taken at T0-T3 were placed in liquid N_2 and finely powdered in a mortar with a pestle. Then, they were treated with 3 ml of 2:1 v/v methanol/chloroform, at 4 °C. Samples were transferred in separate glass vials, where 1 ml of chloroform and 1.2 ml of distilled water were added to form an emulsion. Samples were vortexed and then kept at 4 °C for 40 min. Resulting homogenates were then centrifuged at 10,000 rpm for 20 min, at 4 °C. The upper (hydroalcoholic) and lower (organic) phases were carefully separated. Remaining pellets were re-extracted using half of the solvent volumes, under the same conditions described above; separated fractions were pooled and added to the counterparts from the first extraction. Hydroalcoholic and organic extracts from each kiwifruit sample were dried under a N_2 flow, at 25 °C.

Dried hydroalcoholic and organic extracts were solved as reported in the main text. NMR spectra were recorded at 27 °C on a Bruker AVANCE 600 instrument. ^{1}H spectra were referenced to CH_3 signals of TSP (= 0.00 ppm) in D_2O, and to the residual CHD_2 signal of methanol (set to 3.31 ppm) in $CD_3OD/CDCl_3$. ^{1}H spectra of aqueous extracts were acquired by coadding 256 transients with a recycle delay of 3 s. The residual HDO signal was suppressed using a standard pre-saturation pulse

sequence. Experiments were carried out by using a 45° pulse of 5.0 μs and 32K data points. ^1H spectra of $CD_3OD/CDCl_3$ extracts were obtained using 256 transients, 64K data points, a recycle delay of 3 s and a 90° pulse of 10 μs. The following 2D NMR experiments were performed on organic extracts: i) ^1H–^1H TOtal Correlation SpectroscopY (TOCSY); ii) ^1H–^{13}C Heteronuclear Single Quantum Coherence (HSQC); iii) ^1H–^{13}C Heteronuclear Multiple-Bond Correlation (HMBC). The mixing time for the ^1H–^1H TOCSY was 80 ms. HSQC experiments were performed using a coupling constant $^1J_{C-H}$ of 150 Hz; ^1H–^{13}C HMBC experiments were performed using a delay for the evolution of long-range couplings of 80 ms.

Assignment of ^1H spectra of aqueous extracts was realized as previously reported (Salzano et al., 2018). Diagnostic ^1H-NMR signals identified and quantified various metabolites in aqueous extracts, namely malic acid (MA), citric acid (CA), ascorbic acid (AA), lactic acid (LA), quinic acid (QA), sugars α- and β-glucose (AGLC, and BGLC), sucrose (SUCR), β-fructopyranose (BFRUPY), amino acids Ala, Thr, Glu, Asp, Val, Ile, Trp and γ-aminobutyric acid, and other compounds such as choline (CHN), uridine (URI) and myo-inositol (MI). Assignment of ^1H spectra of kiwifruit organic extracts was based on literature data and 2D experiments (Salzano et al., 2018), allowing identification of poly-unsaturated fatty acids (PUFA), di-unsaturated fatty acids (DUFA), unsaturated fatty acids (UFA), mono-unsaturated fatty acids (MUFA), saturated fatty acids (SFA), phosphatidylcholine (PC), phosphatidylethanolamine (PE), digalactosyldiacylglycerol (DG), stigmasterol (ST), stigmast-7-en-3β-ol (S7), β-sitosterol and campesterol. Since the ^1H-NMR signals from 18-CH_3 group of β-sitosterol and campesterol were completely overlapped, and all the other sterol signals were overlapped with fatty acid chain signals, only the sum of β-sitosterol and campesterol (bSC) was quantified.

Metabolite concentrations were derived from the integral values of the corresponding resonances in ^1H NMR spectra (Salzano et al., 2018). For aqueous extracts, the integral values were normalised to the integral of TSP methyl group and the content of selected metabolites was calculated (in mg/g

or mg/kg of fresh weight - FW). For organic extracts, spectral integrals were normalized to the integral of α-CH_2 groups of all fatty acid chains that were set to 100%. Then, the total content (molar percentage, % mol) of fatty acids of four different types, *i.e.* saturated, mono-unsaturated, di-unsaturated and poly-unsaturated ones, was calculated. The integrals of selected resonances due to sterols, phospholipids and galactolipids were also normalized to the integral of α-CH_2 groups of all fatty acid chains. Quantification of all metabolites at T0-T3 stages is shown in Supplementary Fig. S1 and S2.

Extraction of kiwifruit polyphenolics for LC-UV analysis combined with ESI-IT-MS^n

Extraction of polyphenols from outer pericarp samples (about 2.5 g) taken at different postharvest stages (T0-T3) was carried out as previously described (Salzano et al., 2018). Briefly, fruit samples were finely chopped and treated with 6 ml of 1% v/v acetic acid in acetone in an ultrasonic bath for 15 min, and in a horizontal shaker for 20 min, in the dark. The supernatant was removed and the extraction was repeated twice. For each sample, supernatants were pooled and dried in a LaboRota 4000/HB Efficient rotary evaporator (Heidolph, Schwabach, Germany). Sample extracts were reconstituted in 3% formic acid and then passed through a C18 Sep-Pak cartridge (Waters, Milford, MA), as previously reported (Salzano et al., 2018).

2D-DIGE and 2-DE analysis

Fifty µg of proteins from each biological sample were labeled with 400 pmol of Cy3- or Cy5-dyes (GE Healthcare, UK), using the dye-swapping strategy (Supplementary Tab. S1). A mixture of all biological samples (12 in number) was labeled with Cy2 dye and used as internal standard, according to 2D-DIGE protocol (Alban et al., 2003). Each labeling reaction was performed at 0 °C for 30 min, in the dark, and quenced with 1 mM lysine. Appropriate Cy3- and Cy5-labeled pairs and a Cy2-labeled control were used to generate mixtures. Each mixture was supplemented with 1% v/v IPG buffer (an optimized mixture of carrier ampholytes), pH 3-10 NL (GE Healthcare), 1.4% v/v DeStreak

reagent (GE Healthcare) and 0.2% w/v DTT to reach a final volume of 450 μl in 7 M urea, 2 M thiourea, and 4% w/v CHAPS. The mixtures (150 μg of total protein content) were used for passive hydration of immobilized pH gradient IPG gel strips (24 cm, pH 3-10 NL), in the dark, for 16 h, at 20 °C. Isoelectric focusing (IEF) was carried out with an IPGphor II apparatus (GE Healthcare) up to 80,000 V/h at 20 °C. After IEF, each strip was equilibrated with an equilibration solution composed of 6 M urea, 2% w/v SDS, 20% w/v glycerol, and 0.375 M Tris–HCl (pH 8.8), in the presence of 0.5% w/v DTT, for 15 min, in the dark; then, it was equilibrated in the same buffer containing 4.5% w/v iodacetamide, for another 15 min. Equilibrated IPG strips were transferred onto 12% T polyacrilamide gels to perform the second-dimension SDS-PAGE, using an ETTAN DALT six electrophoresis system (GE Healthcare).

Resulting gels were scanned with a Typhoon 9400 variable mode imager (GE Healthcare) using proper excitation/emission wavelengths for Cy2 (488/520 nm), Cy3 (532/580 nm), and Cy5 (633/670 nm). Gel images were cropped and visualized within the Image-Quant software (GE Healthcare) and analyzed using the DeCyder 5.0 software (GE Healthcare). A DeCyder differential In-gel-Analysis (DIA) module was used for spot detection, background subtraction and pairwise comparison of each sample (Cy3 and Cy5) to the Cy2 mixed standard present in each gel, in order to perform Cy5/Cy2 and Cy3/Cy2 normalization. Then, the DeCyder Biological Variation Analysis (BVA) module was used to simultaneously match all of the protein-spot maps from the gels, and to calculate average abundance ratios and statistical parameters (Student's *t*-test). Differentially represented spots were identified as those having a relative expression ratio ≥1.50 or ≤-1.50, with a p value ≤ 0.05.

Preparative 2-DE was performed using 500 μg of unlabeled proteins from mixed biological samples. Resulting 2-DE gel was stained with Sypro Ruby, according to the manufacturer's instructions (ThermoFisher). After spot matching with the master gel from the analytical assay in the BVA module of DeCyder software, a pick list was generated for spot picking by an Ettan spot robotic picker (GE Healthcare).

References

Alban, A., David, S. O., Bjorkesten, L., Andersson, C., Sloge, E., Lewis, S., et al. (2003). A novel experimental design for comparative two-dimensional gel analysis: Two-dimensional difference gel electrophoresis incorporating a pooled internal standard. Proteomics. 3, 36-44. doi:10.1002/pmic.200390006.

Bevan, M., Bancroft, I., Bent, E., Love, K., Goodman, H., Dean, C., et al. (1998). Analysis of 1.9 Mb of contiguous sequence from chromosome 4 of Arabidopsis thaliana. Nature. 391, 485-488. doi:10.1038/35140.

Bradford, M. M. (1976). A rapid and sensitive method for the quantitation of microgram quantities of protein utilizing the principle of protein-dye binding. Anal. Biochem. 72, 248-254. doi:10.1016/0003-2697(76)90527-3.

Bulley, S. M. (2016). The Kiwifruit Allergome, in The Kiwifruit Genome, eds. R. Testolin, H. W. Huang, A. R. Ferguson (Switzerland: Springer), 219–235. doi:10.1007/978-3-319-32274-2_17.

McGuire, R. G. (1992). Reporting of objective color measurements. HortScience. 27, 1254-1255.

Salzano, A. M., Sobolev, A., Carbone, V., Petriccione, M., Renzone, G., Capitani, D., et al. (2018). A proteometabolomic study of *Actinidia deliciosa* fruit development. J. Proteomics. 172, 11-24. doi:10.1016/j.jprot.2017.11.004.

Wang, W., Scali, M., Vignani, R., Spadafora, A., Sensi, E., Mazzuca, S., et al. (2003). Protein extraction for two-dimensional electrophoresis from olive leaf, a plant tissue containing high levels of interfering compounds. Electrophoresis. 24, 2369-2375. doi:10.1002/elps.200305500.

Supplementary Tab. S1. Experimental design of 2D-DIGE for the analysis of outer pericarp proteins from kiwifruit at different postharvest stages (T0, T1, T2 and T3). Scheme of labeling for protein extracts. Reference mixture was a pool of all biological replicates from all postharvest stages. Biological replicate number is reported in parenthesis.

**Gel**	**Cy2**	**Cy3**	**Cy5**
1	Reference mixture	T0 (1)	T1 (1)
2	Reference mixture	T2 (1)	T3 (1)
3	Reference mixture	T1 (3)	T2 (2)
4	Reference mixture	T3 (2)	T0 (2)
5	Reference mixture	T0 (3)	T2 (3)
6	Reference mixture	T1 (2)	T3 (3)

Supplementary Tab. S3. Differentially represented proteins in the outer pericarp of kiwifruits sampled at different postharvest stages (T0, T1, T2 and T3) as deriving from combined 2D-DIGE/nLC-ESI-LIT-MS/MS analysis. Proteins are listed according to their functional classification (Bevan et al., 1998). Spot number, kiwifruit database accession code, protein name, gene name of the *Arabidopsis thaliana* protein homologue with the highest sequence similarity, corresponding TAIR 10 accession code, corresponding sequence identity (%), Mascot score, theoretical Mr and pI values, number of peptides identified, number of unique peptides identified, and sequence coverage (%) values are listed, together the corresponding fold change values at various stages (T1, T2 and T3 with respect to T0). For *Arabidopsis thaliana* protein homologues present in the TAIR 10 database that showed a sequence similarity lower than 60%, a Blast search against the plant NCBI database was also performed (data not shown).

Spot	Accession - Kiwifruit database	Protein description	Gene name	Accession TAIR 10	Identity (%)	Mascot score	Mr (Da)	pI	PSMs	Σ peptide matches	Σ unique peptide mathes	Sequence coverage (%)	T1 vs T0	T2 vs T0	T3 vs T0
Cell growth/division															
1697	Achn010071	Dynamin, putative	ADL1E	AT3G60190	80.2	123	70231	7.66	3	2	2	5.3			2.18
1756	Achn010071	Dynamin, putative	ADL1E	AT3G60190	80.2	179	70231	7.66	4	3	3	6.5	3.80	6.37	14.00
1207	Achn066661	Transitional endoplasmic reticulum ATPase, putative	AtCDC48C	AT5G03340	93.2	968	89797	5.16	26	19	15	24.9	2.36		2.38
2817	Achn066661	Transitional endoplasmic reticulum ATPase, putative	AtCDC48C	AT5G03340	93.2	361	89797	5.16	6	6	6	8.4	-1.66	-1.87	
Cell structure															
2675	Achn107181	Actin 1	ACT7	AT5G09810	98.7	253	41865	5.31	5	4	4	15.1			-1.65
3575	Achn107181	Actin 1	ACT7	AT5G09810	98.7	157	41865	5.31	5	2	2	11.1		-1.50	
4528	Achn134781	Annexin, putative	ANNAT4	AT2G38750	52.5	127	36009	6.62	3	2	2	10.9	-1.92		
4764	Achn134781	Annexin, putative	ANNAT4	AT2G38750	52.5	138	36009	6.62	2	2	2	8.6			-1.95
3990	Achn031611	Arabinogalactan protein	AT5G11680	AT5G11680	78.6	129	23203	6.65	3	2	2	21.1			-1.71
3996	Achn031611	Arabinogalactan protein	AT5G11680	AT5G11680	78.6	180	23203	6.65	4	3	3	30.9		-1.55	-2.05
1848	Achn262021	Beta-xylosidase 4	XYL4	AT5G64570	71.0	100	83425	8.15	2	2	2	3.2			1.79
1909	Achn262021	Beta-xylosidase 4	XYL4	AT5G64570	71.0	86	83425	8.15	2	2	2	3.4	1.62		
4580	Achn102711	Pectinesterase inhibitor	PMEI2	AT3G17220	36.7	194	30831	4.73	3	3	3	16.9	3.33	2.39	3.80
1849	Achn107321	Pectinesterase-2, putative	PME51	AT5G09760	68.5	113	59590	6.19	2	2	2	5.1		1.70	2.69

3780	Achn150541	Plastid-lipid-associated protein, chloroplastic	AT4G22240	AT4G22240	73.6	300	26708	4.97	6	5	4	25.3	-1.71		
2643	Achn126481	Polygalacturonase-inhibitor protein	PGIP1	AT5G06860	67.5	277	36557	8.86	9	5	5	20.5			2.04
2921	Achn234251	Putative uncharacterized protein P0046B10.2-1 (protein of unknown function DUF642, galactose-binding domain-like)	AT3G08030	AT3G08030	73.2	234	37409	9	10	8	4	13.7			1.62
2356	Achn027511	Tubulin beta-2 chain	TUB5	AT1G20010	75.2	136	44173	4.44	2	2	2	6.4		-2.01	
Disease/defense															
3487	Achn305291	Abscisic stress ripening protein homolog (ABA/WDS induced)	AT1G70810	AT1G70810	38.5	183	20048	5.88	4	4	3	32.4	-1.54	-1.81	
3502	Achn305291	Abscisic stress ripening protein homolog (ABA/WDS induced)	AT1G70810	AT1G70810	38.5	175	20048	5.88	5	5	4	32.4	-1.57	-2.38	
3844	Achn305291	Abscisic stress ripening protein homolog (ABA/WDS induced)	AT1G70810	AT1G70810	38.5	191	20048	5.88	4	4	4	32.4		-1.68	
3976	Achn305291	Abscisic stress ripening protein homolog (ABA/WDS induced)	AT1G70810	AT1G70810	38.5	161	20048	5.88	4	4	3	18.1			-1.84
2214	Achn294821	Alpha-toxin (aerolysin/haemolysin)	AT4G32480	AT4G32480	45.5	225	53572	6.61	11	10	4	10.1			-1.89
2291	Achn294821	Alpha-toxin (aerolysin/haemolysin)	AT4G32480	AT4G32480	45.5	144	53572	6.61	6	4	2	7.7			-1.72
2395	Achn294821	Alpha-toxin (aerolysin/haemolysin)	AT4G32480	AT4G32480	45.5	88	53572	6.61	2	2	2	4.5			-1.59
2905	Achn294821	Alpha-toxin (aerolysin/haemolysin)	AT4G32480	AT4G32480	45.5	219	53572	6.61	4	3	3	9.7		-1.85	-1.95
2291	Achn260011	Catalase-3	CAT2	AT4G35090	74.0	153	52586	6.85	3	3	3	7.3			-1.72
4409	Achn303071	Cbs domain protein (cystathionine beta-synthase)	CBSX3	AT5G10860	66.5	150	23483	7.74	5	3	2	17.1	-2.72	-2.07	-2.47
1111	Achn194541	Chaperone clpb (chaperonin ClpA/B)	DLT1	AT1G74310	82.8	397	105568	5.7	7	6	6	9.1			1.82
1459	Achn194541	Chaperone clpb (chaperonin ClpA/B)	DLT1	AT1G74310	82.8	135	105568	5.7	3	2	2	3.4	1.55		
1232	Achn161991	Chaperone protein clpB (chaperonin ClpA/B)	CLPB3	AT5G15450	85.0	418	120255	6.22	8	6	6	8.5			1.52
1233	Achn161991	Chaperone protein clpB (chaperonin ClpA/B)	CLPB3	AT5G15450	85.0	371	120255	6.22	7	7	7	8.2	1.56		1.94
3735	Achn340321	Chitinase	HCHIB	AT3G12500	70.8	139	58640	8.76	5	4	2	5	-2.69		
2557	Achn343961	Dehydrin 2	AT1G20440	AT1G20440	41.1	119	23322	5.17	3	3	2	13.7	-1.80		
2817	Achn343961	Dehydrin 2	AT1G20440	AT1G20440	41.1	120	23322	5.17	2	2	2	14.6	-1.66	-1.87	
3847	Achn351281	Glutathione S-transferase	GSTL3	AT5G02790	69.6	93	27640	5.6	2	2	2	11.7	-1.69	-1.82	-2.54
1657	Achn177881	Heat shock 70 kDa protein C	BIP2	AT5G42020	88.7	847	70680	5.03	18	13	12	29.2	1.67		
1507	Achn079561	Heat shock protein 90-2	Hsp81	AT5G56000	91.7	412	80452	4.99	8	6	6	12.4			2.34
1047	Achn022471	Kiwellin (Barwin-like endoglucanase)	AT5G60615	AT5G60615	27.6	133	23040	5.68	3	2	2	18.3			1.71
1362	Achn022471	Kiwellin (Barwin-like endoglucanase)	AT5G60615	AT5G60615	27.6	201	23040	5.68	3	3	3	26.8		1.60	1.93
1467	Achn022471	Kiwellin (Barwin-like endoglucanase)	AT5G60615	AT5G60615	27.6	158	23040	5.68	3	3	3	26.8			-1.62
1728	Achn022471	Kiwellin (Barwin-like endoglucanase)	AT5G60615	AT5G60615	27.6	195	23040	5.68	3	3	3	27.2			-1.82

2004	Achn022471	Kiwellin (Barwin-like endoglucanase)	AT5G60615	AT5G60615	27.6	88	23040	5.68	2	2	2	14.1	1.72		1.83
2093	Achn022471	Kiwellin (Barwin-like endoglucanase)	AT5G60615	AT5G60615	27.6	268	23040	5.68	5	5	4	28.6	2.28	1.59	2.49
2141	Achn022471	Kiwellin (Barwin-like endoglucanase)	AT5G60615	AT5G60615	27.6	268	23040	5.68	4	4	4	33.8			1.54
2332	Achn022471	Kiwellin (Barwin-like endoglucanase)	AT5G60615	AT5G60615	27.6	220	23040	5.68	3	3	3	25.4			1.63
2339	Achn022471	Kiwellin (Barwin-like endoglucanase)	AT5G60615	AT5G60615	27.6	173	23040	5.68	4	3	3	29.6			1.95
2398	Achn022471	Kiwellin (Barwin-like endoglucanase)	AT5G60615	AT5G60615	27.6	249	23040	5.68	6	4	4	34.3			-1.71
2606	Achn022471	Kiwellin (Barwin-like endoglucanase)	AT5G60615	AT5G60615	27.6	235	23040	5.68	4	4	4	33.3			1.94
2904	Achn022471	Kiwellin (Barwin-like endoglucanase)	AT5G60615	AT5G60615	27.6	398	23040	5.68	11	10	5	49.3			1.72
2905	Achn022471	Kiwellin (Barwin-like endoglucanase)	AT5G60615	AT5G60615	27.6	142	23040	5.68	3	2	2	21.1		-1.85	-1.95
3196	Achn022471	Kiwellin (Barwin-like endoglucanase)	AT5G60615	AT5G60615	27.6	328	23040	5.68	6	6	5	41.3		2.41	4.16
3281	Achn022471	Kiwellin (Barwin-like endoglucanase)	AT5G60615	AT5G60615	27.6	126	23040	5.68	2	2	2	12.2			1.53
3284	Achn022471	Kiwellin (Barwin-like endoglucanase)	AT5G60615	AT5G60615	27.6	230	23040	5.68	3	3	3	28.2			-1.56
3452	Achn022471	Kiwellin (Barwin-like endoglucanase)	AT5G60615	AT5G60615	27.6	140	23040	5.68	4	2	2	20.2	1.79	1.74	
3685	Achn022471	Kiwellin (Barwin-like endoglucanase)	AT5G60615	AT5G60615	27.6	206	23040	5.68	3	3	3	27.2			-1.81
3694	Achn022471	Kiwellin (Barwin-like endoglucanase)	AT5G60615	AT5G60615	27.6	88	23040	5.68	2	2	2	15		-1.90	-1.55
3714	Achn022471	Kiwellin (Barwin-like endoglucanase)	AT5G60615	AT5G60615	27.6	200	23040	5.68	5	2	2	30.5			-1.61
3780	Achn022471	Kiwellin (Barwin-like endoglucanase)	AT5G60615	AT5G60615	27.6	135	23040	5.68	4	3	2	27.7	-1.71		
3793	Achn022471	Kiwellin (Barwin-like endoglucanase)	AT5G60615	AT5G60615	27.6	201	23040	5.68	3	3	3	26.3			-1.66
3823	Achn022471	Kiwellin (Barwin-like endoglucanase)	AT5G60615	AT5G60615	27.6	363	23040	5.68	8	6	5	44.6			-1.67
3847	Achn022471	Kiwellin (Barwin-like endoglucanase)	AT5G60615	AT5G60615	27.6	106	23040	5.68	3	3	2	12.7	-1.69	-1.82	-2.54
3857	Achn022471	Kiwellin (Barwin-like endoglucanase)	AT5G60615	AT5G60615	27.6	255	23040	5.68	5	5	4	33.3			-1.73
3874	Achn022471	Kiwellin (Barwin-like endoglucanase)	AT5G60615	AT5G60615	27.6	383	23040	5.68	20	17	6	55.4			-1.78
3880	Achn022471	Kiwellin (Barwin-like endoglucanase)	AT5G60615	AT5G60615	27.6	335	23040	5.68	11	10	5	42.3			-1.72
3894	Achn022471	Kiwellin (Barwin-like endoglucanase)	AT5G60615	AT5G60615	27.6	622	23040	5.68	27	25	9	62.9			-1.70
3896	Achn022471	Kiwellin (Barwin-like endoglucanase)	AT5G60615	AT5G60615	27.6	420	23040	5.68	11	10	6	48.4	-1.73		-2.33
3903	Achn022471	Kiwellin (Barwin-like endoglucanase)	AT5G60615	AT5G60615	27.6	325	23040	5.68	9	9	6	48.4			-2.06
3913	Achn022471	Kiwellin (Barwin-like endoglucanase)	AT5G60615	AT5G60615	27.6	293	23040	5.68	7	6	5	34.7	-1.57		
3941	Achn022471	Kiwellin (Barwin-like endoglucanase)	AT5G60615	AT5G60615	27.6	312	23040	5.68	20	18	5	38.5	-1.51		
3958	Achn022471	Kiwellin (Barwin-like endoglucanase)	AT5G60615	AT5G60615	27.6	352	23040	5.68	6	6	5	41.3			-1.64
3976	Achn022471	Kiwellin (Barwin-like endoglucanase)	AT5G60615	AT5G60615	27.6	379	23040	5.68	6	6	5	39.9			-1.84
3983	Achn022471	Kiwellin (Barwin-like endoglucanase)	AT5G60615	AT5G60615	27.6	457	23040	5.68	13	12	7	54.9			-1.60

3990	Achn022471	Kiwellin (Barwin-like endoglucanase)	AT5G60615	AT5G60615	27.6	331	23040	5.68	5	4	4	39.9			-1.71
3991	Achn022471	Kiwellin (Barwin-like endoglucanase)	AT5G60615	AT5G60615	27.6	209	23040	5.68	3	3	3	28.2	-1.58		
4051	Achn022471	Kiwellin (Barwin-like endoglucanase)	AT5G60615	AT5G60615	27.6	243	23040	5.68	7	7	4	27.2	-1.63		
4062	Achn022471	Kiwellin (Barwin-like endoglucanase)	AT5G60615	AT5G60615	27.6	82	23040	5.68	2	2	2	15	-1.81		
4452	Achn022471	Kiwellin (Barwin-like endoglucanase)	AT5G60615	AT5G60615	27.6	325	23040	5.68	6	3	3	42.3			-1.70
4551	Achn022471	Kiwellin (Barwin-like endoglucanase)	AT5G60615	AT5G60615	27.6	154	23040	5.68	3	3	3	20.7	-2.16	-1.86	-2.68
4717	Achn022471	Kiwellin (Barwin-like endoglucanase)	AT5G60615	AT5G60615	27.6	153	23040	5.68	2	2	2	19.7			-1.87
4787	Achn022471	Kiwellin (Barwin-like endoglucanase)	AT5G60615	AT5G60615	27.6	350	23040	5.68	10	7	5	48.8			-1.98
4806	Achn022471	Kiwellin (Barwin-like endoglucanase)	AT5G60615	AT5G60615	27.6	168	23040	5.68	3	3	3	20.7			-3.18
4807	Achn022471	Kiwellin (Barwin-like endoglucanase)	AT5G60615	AT5G60615	27.6	312	23040	5.68	7	5	4	33.8			-2.65
4711	Achn220691	Lectin (ricin B lectin)	EULS3	AT2G39050	46.0	87	46396	6.3	3	3	2	6			1.51
1715	Achn277251	Major latex-like protein (Bet v I)	MLP28	AT1G70830	42.9	97	17401	4.69	2	2	2	14.7		-1.57	
1754	Achn277251	Major latex-like protein (Bet v I)	MLP28	AT1G70830	42.9	136	17401	4.69	2	2	2	14.7		-1.56	
2557	Achn277251	Major latex-like protein (Bet v I)	MLP28	AT1G70830	42.9	139	17401	4.69	3	3	2	14.7	-1.80		
2675	Achn277251	Major latex-like protein (Bet v I)	MLP28	AT1G70830	42.9	141	17401	4.69	2	2	2	14.7			-1.65
2701	Achn277251	Major latex-like protein (Bet v I)	MLP28	AT1G70830	42.9	119	17401	4.69	6	6	2	14.7	-1.85	-2.30	
2817	Achn277251	Major latex-like protein (Bet v I)	MLP28	AT1G70830	42.9	122	17401	4.69	4	3	2	14.7	-1.66	-1.87	
2836	Achn277251	Major latex-like protein (Bet v I)	MLP28	AT1G70830	42.9	96	17401	4.69	2	2	2	14.7			2.06
2921	Achn277251	Major latex-like protein (Bet v I)	MLP28	AT1G70830	42.9	138	17401	4.69	2	2	2	14.7			1.62
3079	Achn277251	Major latex-like protein (Bet v I)	MLP28	AT1G70830	42.9	117	17401	4.69	2	2	2	14.7			-1.91
3166	Achn277251	Major latex-like protein (Bet v I)	MLP28	AT1G70830	42.9	148	17401	4.69	12	11	2	14.7			1.80
3201	Achn277251	Major latex-like protein (Bet v I)	MLP28	AT1G70830	42.9	129	17401	4.69	4	3	2	14.7			1.90
3568	Achn277251	Major latex-like protein (Bet v I)	MLP28	AT1G70830	42.9	173	17401	4.69	5	5	3	28		-1.91	-1.83
3571	Achn277251	Major latex-like protein (Bet v I)	MLP28	AT1G70830	42.9	106	17401	4.69	2	2	2	14.7		-1.90	-2.32
3575	Achn277251	Major latex-like protein (Bet v I)	MLP28	AT1G70830	42.9	115	17401	4.69	3	3	2	14.7		-1.50	
3685	Achn277251	Major latex-like protein (Bet v I)	MLP28	AT1G70830	42.9	132	17401	4.69	3	3	2	14.7			-1.81
3780	Achn277251	Major latex-like protein (Bet v I)	MLP28	AT1G70830	42.9	98	17401	4.69	2	2	2	14.7	-1.71		
4051	Achn277251	Major latex-like protein (Bet v I)	MLP28	AT1G70830	42.9	128	17401	4.69	6	6	2	14.7	-1.63		
4580	Achn277251	Major latex-like protein (Bet v I)	MLP28	AT1G70830	42.9	127	17401	4.69	2	2	2	14.7	3.33	2.39	3.80
4787	Achn277251	Major latex-like protein (Bet v I)	MLP28	AT1G70830	42.9	206	17401	4.69	6	6	4	30			-1.98
4805	Achn277251	Major latex-like protein (Bet v I)	MLP28	AT1G70830	42.9	185	17401	4.69	12	12	3	18.7			1.92

4827	Achn277251	Major latex-like protein (Bet v I)	MLP28	AT1G70830	42.9	363	17401	4.69	15	13	5	48.7			2.41
1938	Achn075231	Monodehydroascorbate reductase	MDAR1	AT3G52880	70.7	142	60414	6.27	3	2	2	7.1		1.71	2.75
2657	Achn075231	Monodehydroascorbate reductase	MDAR1	AT3G52880	70.7	337	60414	6.27	8	7	5	12.2			2.15
2682	Achn075231	Monodehydroascorbate reductase	MDAR1	AT3G52880	70.7	291	60414	6.27	7	6	4	12.2			1.65
2693	Achn075231	Monodehydroascorbate reductase	MDAR1	AT3G52880	70.7	446	60414	6.27	7	7	7	16.9			1.75
3913	Achn332771	NtPRp27-like protein	AT2G15220	AT2G15220	60.9	317	34764	5.42	16	15	6	20.1	-1.57		
1956	Achn353051	Protein disulfide isomerase-like protein	PDIL1-1	AT1G21750	64.6	496	55650	5.08	12	8	8	23.5	-1.64	-2.01	
3983	Achn259181	Putative glutathione S-transferase	GSTF7	AT1G02920	59.0	241	15107	5.44	6	5	5	36.6			-1.60
3990	Achn259181	Putative glutathione S-transferase	GSTF7	AT1G02920	59.0	216	15107	5.44	8	6	3	26.9			-1.71
3991	Achn259181	Putative glutathione S-transferase	GSTF7	AT1G02920	59.0	154	15107	5.44	3	3	3	17.2	-1.58		
3996	Achn259181	Putative glutathione S-transferase	GSTF7	AT1G02920	59.0	184	15107	5.44	9	6	3	23.9		-1.55	-2.05
4020	Achn259181	Putative glutathione S-transferase	GSTF7	AT1G02920	59.0	138	15107	5.44	3	2	2	26.1			-1.79
1761	Achn089541	Stress-induced-phosphoprotein	Hop3	AT4G12400	74.1	264	65309	5.82	6	5	5	11.7		-1.77	
4797	Achn052701	Superoxide dismutase [Cu-Zn]	CSD2	AT2G28190	89.7	247	14290	5.33	5	5	4	39.4			-3.57
3685	Achn319351	Superoxide dismutase [Mn-Fe]	FSD2	AT5G51100	71.3	108	34554	5.36	3	2	2	11.8			-1.81
1052	Achn132631	Thaumatin-like protein	OSM34	AT4G11650	68.9	84	25221	8.29	2	2	2	10.2			-1.69
1108	Achn332061	Thaumatin-like protein	OSM34	AT4G11650	63.1	87	34834	8.38	2	2	2	4.5			2.01
1346	Achn332061	Thaumatin-like protein	OSM34	AT4G11650	63.1	83	34834	8.38	2	2	2	4.5		1.54	1.68
1486	Achn132621	Thaumatin-like protein	OSM34	AT4G11650	69.3	126	26621	7.8	2	2	2	9.7	2.02	2.71	
1528	Achn132631	Thaumatin-like protein	OSM34	AT4G11650	68.9	93	25221	8.29	2	2	2	11.6			-1.61
1581	Achn132631	Thaumatin-like protein	OSM34	AT4G11650	68.9	99	25221	8.29	2	2	2	11.6	1.92	2.61	4.31
1828	Achn132631	Thaumatin-like protein	OSM34	AT4G11650	68.9	99	25221	8.29	2	2	2	11.6	3.12	5.27	15.06
2039	Achn332061	Thaumatin-like protein	OSM34	AT4G11650	63.1	124	34834	8.38	3	2	2	4.5			1.57
2542	Achn132621	Thaumatin-like protein	OSM34	AT4G11650	69.3	103	26621	7.8	2	2	2	9.7		1.61	2.10
2553	Achn132621	Thaumatin-like protein	OSM34	AT4G11650	69.3	142	26621	7.8	2	2	2	10.2	1.93		
3027	Achn132631	Thaumatin-like protein	OSM34	AT4G11650	68.9	193	25221	8.29	3	3	3	17.3			-1.66
3110	Achn132631	Thaumatin-like protein	OSM34	AT4G11650	68.9	89	25221	8.29	2	2	2	11.6	1.63		
3950	Achn145801	Thaumatin-like protein	OSM34	AT4G11650	68.6	269	24906	4.84	4	4	4	21.8			-2.46
3964	Achn132631	Thaumatin-like protein	OSM34	AT4G11650	68.9	248	25221	8.29	5	5	4	21.3			-1.65
3990	Achn132631	Thaumatin-like protein	OSM34	AT4G11650	68.9	250	25221	8.29	4	3	3	21.3			-1.71
4003	Achn132631	Thaumatin-like protein	OSM34	AT4G11650	68.9	405	25221	8.29	27	21	6	28.4			-1.90

4005	Achn132631	Thaumatin-like protein	OSM34	AT4G11650	68.9	355	25221	8.29	21	17	5	27.1			-1.88
4020	Achn132631	Thaumatin-like protein	OSM34	AT4G11650	68.9	240	25221	8.29	11	8	4	23.1			-1.79
4052	Achn132631	Thaumatin-like protein	OSM34	AT4G11650	68.9	220	25221	8.29	5	3	3	22.7			-1.84
4062	Achn132631	Thaumatin-like protein	OSM34	AT4G11650	68.9	122	25221	8.29	3	2	2	12.9	-1.81		
4107	Achn132631	Thaumatin-like protein	OSM34	AT4G11650	68.9	437	25221	8.29	21	17	6	28.4			-1.57
4527	Achn132631	Thaumatin-like protein	OSM34	AT4G11650	68.9	112	25221	8.29	2	2	2	11.6	-2.31		-3.23
4711	Achn132631	Thaumatin-like protein	OSM34	AT4G11650	68.9	122	25221	8.29	2	2	2	10.2			1.51
4305	Achn007751	Universal stress protein 1	AT3G53990	AT3G53990	69.6	213	22806	6.12	6	2	2	22.9			1.60
Energy															
3548	Achn223971	3-hydroxyisobutyrate dehydrogenase, putative	AT1G71170	AT1G71170	60.0	240	29242	5.28	4	4	4	16.3		-1.58	-1.88
1184	Achn079831	3-isopropylmalate dehydratase large subunit	ACO3	AT2G05710	82.6	393	143020	6.19	9	6	6	7.6			1.53
1196	Achn079831	3-isopropylmalate dehydratase large subunit	ACO3	AT2G05710	82.6	557	143020	6.19	10	6	6	8.4	1.53		1.74
1198	Achn079831	3-isopropylmalate dehydratase large subunit	ACO3	AT2G05710	82.6	482	143020	6.19	8	7	7	7	1.51		1.59
2380	Achn321141	6-phosphogluconate dehydrogenase, decarboxylating	AT3G02360	AT3G02360	89.3	255	50902	6.37	4	4	4	10.2			2.31
2386	Achn321141	6-phosphogluconate dehydrogenase, decarboxylating	AT3G02360	AT3G02360	89.3	213	50902	6.37	4	3	3	10.4		1.69	2.28
2399	Achn321141	6-phosphogluconate dehydrogenase, decarboxylating	AT3G02360	AT3G02360	89.3	362	50902	6.37	6	5	5	17.5			1.68
2419	Achn321141	6-phosphogluconate dehydrogenase, decarboxylating	AT3G02360	AT3G02360	89.3	342	50902	6.37	7	5	5	18.6			1.57
3714	Achn346601	6-phosphogluconolactonase	AT5G24400	AT5G24400	60.5	235	28744	5.19	5	5	4	19.3			-1.61
3721	Achn346601	6-phosphogluconolactonase	AT5G24400	AT5G24400	60.5	110	28744	5.19	2	2	2	7.7			-1.60
1198	Achn353581	Aconitate hydratase 2	ACO3	AT2G05710	75.5	396	98084	6.37	7	4	4	10.4	1.51		1.59
1210	Achn353581	Aconitate hydratase 2	ACO3	AT2G05710	75.5	329	98084	6.37	6	6	5	8.7		1.52	1.58
1212	Achn353581	Aconitate hydratase 2	ACO3	AT2G05710	75.5	371	98084	6.37	8	7	5	9.7	1.65	1.51	1.68
1446	Achn115071	Aldehyde dehydrogenase	ALDH7B4	AT1G54100	69.3	130	45820	5.55	2	2	2	5.9	1.65		2.01
1580	Achn022321	Aldehyde dehydrogenase	ALDH2B4	AT3G48000	83.5	115	52262	6.29	2	2	2	6.1	2.17	1.59	2.23
2174	Achn115071	Aldehyde dehydrogenase	ALDH7B4	AT1G54100	69.3	150	45820	5.55	3	3	3	8			1.64
2276	Achn022321	Aldehyde dehydrogenase	ALDH2B6	AT3G48000	83.5	205	52262	6.29	4	3	3	11.4		-1.60	-1.65
2291	Achn022321	Aldehyde dehydrogenase	ALDH2B7	AT3G48000	83.5	214	52262	6.29	6	4	4	13.3			-1.72
2116	Achn235801	Dihydrolipoyl dehydrogenase	mtLPD1	AT1G48030	85.5	233	60945	5.64	5	5	4	10			1.69
2577	Achn354501	Enolase, putative	LOS2	AT2G36530	74.6	142	62299	5.8	2	2	2	5.3			1.59
2335	Achn086741	Enolase	LOS2	AT2G36530	81.0	465	45347	5.2	9	8	7	21.5			1.72

2338	Achn086741	Enolase	LOS2	AT2G36530	81.0	471	45347	5.2	11	9	7	21.8			1.52
2339	Achn086741	Enolase	LOS2	AT2G36530	81.0	595	45347	5.2	11	9	8	29.8			1.95
2583	Achn086741	Enolase	LOS2	AT2G36530	81.0	165	45347	5.2	4	4	4	13.6			1.56
2809	Achn044851	Fructose-bisphosphate aldolase 2	FBA6	AT2G36460	83.5	520	39529	7.01	24	14	9	31.7			1.53
2837	Achn044851	Fructose-bisphosphate aldolase 2	FBA6	AT2G36460	83.5	438	39529	7.01	16	14	7	21.8			1.50
2885	Achn044851	Fructose-bisphosphate aldolase 2	FBA6	AT2G36460	83.5	75	39529	7.01	2	2	2	6.6			1.60
2094	Achn067501	Fructose-bisphosphate aldolase 3	FBA2	AT4G38970	86.3	181	43556	8.62	3	3	3	9			1.69
3153	Achn282911	Fructose-bisphosphate aldolase	FBA3	AT2G01140	79.5	93	67338	7.55	2	2	2	4.5			1.81
1940	Achn014461	Glucose-6-phosphate isomerase	PGIC	AT5G42740	66.5	113	54141	9.07	3	2	2	6.2			1.52
2214	Achn349471	Glyceraldehyde 3-phosphate dehydrogenase, putative	GAPC2	AT1G13440	80.3	197	35989	8.26	3	3	3	11.5			-1.89
2380	Achn323081	Isocitrate dehydrogenase [NADP]	CICDH	AT1G65930	90.2	95	46839	6.73	2	2	2	6.3			2.31
2522	Achn323081	Isocitrate dehydrogenase [NADP]	CICDH	AT1G65930	90.2	581	46839	6.73	20	16	9	29.2	1.74	1.61	2.65
2523	Achn323081	Isocitrate dehydrogenase [NADP]	CICDH	AT1G65930	90.2	367	46839	6.73	6	6	6	18.6			2.25
2528	Achn323081	Isocitrate dehydrogenase [NADP]	CICDH	AT1G65930	90.2	296	46839	6.73	5	5	5	14.3	1.92	2.09	2.72
2679	Achn118341	Isocitrate dehydrogenase [NADP]	AT5G14590	AT5G14590	59.3	99	54860	9.04	2	2	2	5.4			1.56
1802	Achn312431	Malic enzyme	NADP-ME3	AT5G25880	70.1	468	58011	7.16	13	7	6	17.1			1.81
1815	Achn312431	Malic enzyme	NADP-ME3	AT5G25880	70.1	313	58011	7.16	9	5	4	13.4		1.60	2.13
1878	Achn312431	Malic enzyme	NADP-ME3	AT5G25880	70.1	131	58011	7.16	3	2	2	6.5	-1.70		
1554	Achn171131	NADH-ubiquinone oxidoreductase subunit	AT5G37510	AT5G37510	75.2	105	33301	6.18	2	2	2	6.3	2.04		2.05
1413	Achn171121	NADH-ubiquinone oxidoreductase subunit	AT5G37510	AT5G37510	83.4	113	50420	6.98	2	2	2	5.1	1.71		
1508	Achn239151	NADH-ubiquinone oxidoreductase subunit	AT5G37510	AT5G37510	82.0	131	66841	5.97	2	2	2	4.1	2.22	2.20	
1939	Achn339391	Oxalyl-CoA decarboxylase	AT5G17380	AT5G17380	71.9	577	56483	6.58	14	11	9	26.1			1.54
3548	Achn210541	Oxygen-evolving enhancer protein 1 of photosystem II	PSBO1	AT5G66570	86.1	289	38616	5.59	5	4	4	19.3		-1.58	-1.88
3571	Achn210541	Oxygen-evolving enhancer protein 1 of photosystem II	PSBO1	AT5G66570	86.1	261	38616	5.59	4	4	4	17.4		-1.90	-2.32
1556	Achn041831	Phosphoenolpyruvate carboxykinase [ATP], putative	PCK1	AT4G37870	77.6	172	79298	7.12	3	3	3	5.6	1.65	1.75	1.62
935	Achn277511	Phosphoenolpyruvate carboxylase, putative	PEPC	AT3G14940	88.8	148	111040	5.8	3	3	3	3.3	1.69	1.71	1.94
2169	Achn130681	Phosphofructokinase, putative	PFK3	AT4G26270	77.3	131	57524	8.2	2	2	2	5.3			1.82
1845	Achn284661	Phosphoglucomutase, putative	PGM2	AT1G70730	78.9	204	60490	5.46	3	2	2	6.5	1.76		2.34
1846	Achn284661	Phosphoglucomutase, putative	PGM2	AT1G70730	78.9	261	60490	5.46	4	4	4	7.9			1.75
1851	Achn284661	Phosphoglucomutase, putative	PGM2	AT1G70730	78.9	240	60490	5.46	6	4	4	10.1			1.54

1852	Achn284661	Phosphoglucomutase, putative	PGM2	AT1G70730	78.9	245	60490	5.46	4	3	3	10.3			1.86
1853	Achn284661	Phosphoglucomutase, putative	PGM2	AT1G70730	78.9	244	60490	5.46	5	4	4	11.9	1.53		2.26
1858	Achn284661	Phosphoglucomutase, putative	PGM2	AT1G70730	78.9	115	60490	5.46	2	2	2	4.3	1.70		2.13
1909	Achn284661	Phosphoglucomutase, putative	PGM2	AT1G70730	78.9	126	60490	5.46	2	2	2	4.9	1.62		
2094	Achn005301	Phosphoglycerate kinase	PGK	AT1G79550	88.0	119	45698	5.83	2	2	2	6.3			1.69
2116	Achn305841	Phosphoglycerate kinase	PGK	AT1G79550	88.0	119	48655	6.11	2	2	2	5.9			1.69
2835	Achn005301	Phosphoglycerate kinase	PGK	AT1G79550	88.0	727	45698	5.83	32	24	11	38.4			1.74
2836	Achn005301	Phosphoglycerate kinase	PGK	AT1G79550	88.0	356	45698	5.83	8	4	3	17.7			2.06
1843	Achn133211	Putative 2,3-bisphosphoglycerate-independent phosphoglycerate mutase	iPGAM1	AT1G09780	84.2	371	96323	6.44	12	10	6	8.8			1.67
1844	Achn133211	Putative 2,3-bisphosphoglycerate-independent phosphoglycerate mutase	iPGAM1	AT1G09780	84.2	473	96323	6.44	14	11	9	14.1			1.73
1848	Achn133211	Putative 2,3-bisphosphoglycerate-independent phosphoglycerate mutase	iPGAM1	AT1G09780	84.2	75	96323	6.44	2	2	2	2			1.79
1858	Achn133211	Putative 2,3-bisphosphoglycerate-independent phosphoglycerate mutase	iPGAM1	AT1G09780	84.2	261	96323	6.44	6	5	5	5	1.70		2.13
1849	Achn210351	Putative 2,3-bisphosphoglycerate-independent phosphoglycerate mutase	iPGAM1	AT1G09780	74.2	141	55249	5.74	2	2	2	4.8		1.70	2.69
1935	Achn036401	Pyruvate decarboxylase 2	PDC4	AT5G01320	81.1	297	69258	5.96	6	2	2	12.4	2.81	1.96	3.98
1580	Achn071161	Pyruvate kinase	AT5G08570	AT5G08570	88.6	90	42345	6.95	2	2	2	5.4	2.17	1.59	2.23
1637	Achn071161	Pyruvate kinase	AT5G08570	AT5G08570	88.6	260	42345	6.95	4	4	4	15.5		1.54	1.65
2052	Achn071161	Pyruvate kinase	AT5G08570	AT5G08570	88.6	84	42345	6.95	3	3	2	5.4	1.59		1.96
2074	Achn071161	Pyruvate kinase	AT5G08570	AT5G08570	88.6	195	42345	6.95	4	4	4	13.4			1.92
2276	Achn008791	Ribulose bisphosphate carboxylase large chain	RBCL	ATCG00490	95.5	95	58998	6.54	2	2	2	3.9		-1.60	-1.65
4995	Achn374161	Ribulose bisphosphate carboxylase small chain	RBCS1A	AT1G67090	45.6	170	14707	9.4	5	3	2	19.1		-2.54	
935	Achn379981	Succinate dehydrogenase subunit A	SDH1-1	AT5G66760	73.4	95	56366	8.85	2	2	2	4.6	1.69	1.71	1.94
1846	Achn379981	Succinate dehydrogenase subunit A	SDH1-1	AT5G66760	73.4	122	56366	8.85	2	2	2	4.6			1.75
1579	Achn297611	Transketolase, putative	TKL-2	AT2G45290	80.0	488	76602	6.28	12	7	6	13.8			-1.69
1567	Achn215591	Transketolase, putative	AT3G60750	AT3G60750	83.7	327	69027	6.59	8	8	6	11.2			-1.55
1577	Achn215591	Transketolase, putative	AT3G60750	AT3G60750	83.7	350	69027	6.59	8	7	6	12.7			-1.65
3768	Achn340601	Triosephosphate isomerase	TIM	AT2G21170	78.5	146	33287	7.62	3	3	3	7.8	-2.24		
3791	Achn203191	Triosephosphate isomerase	TPI	AT3G55440	82.2	128	30606	6.86	2	2	2	9.3	2.19	1.91	2.56
Metabolism															
2679	Achn072171	3-ketoacyl-CoA thiolase	ACAT2	AT5G48230	76.2	101	40254	5.43	2	2	2	5.7			1.56

1261	Achn238811	Alpha-glucan phosphorylase	AT3G46970	AT3G46970	83.1	563	102393	6.11	11	9	9	16.9	1.56		1.60
1262	Achn238811	Alpha-glucan phosphorylase	AT3G46970	AT3G46970	83.1	210	102393	6.11	5	4	3	6.1	1.76		1.68
1273	Achn238811	Alpha-glucan phosphorylase	AT3G46970	AT3G46970	83.1	156	102393	6.11	3	2	2	4.6	1.84		1.94
1052	Achn177341	Alpha glucosidase-like protein	AT3G23640	AT3G23640	59.5	105	44054	5.52	2	2	2	5.4			-1.69
1055	Achn177341	Alpha glucosidase-like protein	AT3G23640	AT3G23640	59.5	129	44054	5.52	3	2	2	9.2			-1.62
1054	Achn177331	Alpha glucosidase-like protein	AT3G23640	AT3G23640	71.3	103	31669	4.95	2	2	2	7.8			-1.51
2004	Achn122601	Anthranilate phosphoribosyltransferase. putative	AT1G70570	AT1G70570	63.6	157	61687	6.59	4	2	2	8.1	1.72		1.83
3084	Achn224791	Cysteine synthase	OASA1	AT4G14880	82.2	149	77439	4.95	5	4	3	5.2			1.87
3153	Achn224791	Cysteine synthase	OASA1	AT4G14880	82.2	150	77439	4.95	3	3	3	5.2			1.81
1923	Achn299571	D-3-phosphoglycerate dehydrogenase	PGDH1	AT4G34200	79.0	204	80153	5.43	4	2	2	6		1.57	
4062	Achn175371	Dihydrolipoyl dehydrogenase	LPD1	AT3G16950	73.1	206	81578	7.96	4	2	2	7.6	-1.81		
1938	Achn179321	Dihydropyrimidinase	PYD2	AT5G12200	71.7	250	53466	5.52	5	3	3	8.7		1.71	2.75
1946	Achn179321	Dihydropyrimidinase	PYD2	AT5G12200	71.7	220	53466	5.52	6	4	2	8.7	1.80	1.98	3.49
3964	Achn176961	Flavoprotein wrbA	AT4G27270	AT4G27270	92.2	178	22275	5.85	3	3	3	20.2			-1.65
2862	Achn269381	Glutamine synthetase	GLN1-1	AT5G37600	74.4	224	34871	6.41	8	5	4	15.2			1.62
1413	Achn348911	Glycogen debranching enzyme	ISA3	AT4G09020	55.8	94	78517	8.57	2	2	2	3.9	1.71		
2090	Achn367481	Ketol-acid reductoisomerase	AT3G58610	AT3G58610	88.8	205	63901	6.54	5	5	4	9.8			2.24
2719	Achn233891	L-threonine 3-dehydrogenase	SDH	AT5G51970	86.6	388	30298	5.68	9	8	6	31.8		-1.85	
2542	Achn090001	Ornithine aminotransferase	DELTA-OAT	AT5G46180	64.2	376	52823	8.15	7	6	6	20.6		1.61	2.10
2553	Achn090001	Ornithine aminotransferase	DELTA-OAT	AT5G46180	64.2	109	52823	8.15	3	2	2	6.2	1.93		
2090	Achn006391	Phosphoglycerate dehydrogenase	PGDH1	AT4G34200	81.8	147	62421	6.27	3	2	2	6.6			2.24
2094	Achn366051	Putative aldehyde dehydrogenase	ALDH12A1	AT5G62530	81.4	181	64095	7.62	4	4	4	7.8			1.69
2987	Achn377261	Putative zinc-containing alcohol dehydrogenase (oxidoreductase);	AT4G13010	AT4G13010	78.4	299	34518	9.01	6	5	4	13.4			1.55
2978	Achn080991	Quinone oxidoreductase, putative	AT5G61510	AT5G61510	80.7	202	21397	5.56	9	8	3	22.2			1.54
3026	Achn217311	Quinone oxidoreductase-like protein (alcohol dehydrogenase superfamily, zinc-type)	AT3G03080	AT3G03080	67.1	328	35397	5.47	9	7	6	21.6			1.64
3227	Achn004131	Uricase	AT2G26230	AT2G26230	71.8	307	33088	8.56	5	5	5	21	1.67	1.71	2.61
Protein destination and storage															
2454	Achn182781	26S proteasome non-ATPase regulatory subunit	RPN6	AT1G29150	84.4	113	47537	6.01	2	2	2	6.4			1.67
3281	Achn026231	26S proteasome regulatory subunit	RPN11	AT5G23540	95.1	101	34539	6.18	2	2	2	8.7			1.53
2060	Achn231381	3-ketoacyl-CoA synthase	AT1G07750	AT1G07750	45.3	136	37482	6.85	2	2	2	7.3			2.00

3084	Achn231381	3-ketoacyl-CoA synthase	AT1G07750	AT1G07750	45.3	167	37482	6.85	3	3	3	10.2			1.87
3152	Achn231381	3-ketoacyl-CoA synthase	AT1G07750	AT1G07750	45.3	619	37482	6.85	33	19	9	35.7	1.86	1.84	3.46
3153	Achn231381	3-ketoacyl-CoA synthase	AT1G07750	AT1G07750	45.3	112	37482	6.85	3	2	2	7			1.81
3164	Achn231381	3-ketoacyl-CoA synthase	AT1G07750	AT1G07750	45.3	657	37482	6.85	43	32	12	37.1	2.14		2.98
3179	Achn231381	3-ketoacyl-CoA synthase	AT1G07750	AT1G07750	45.3	514	37482	6.85	17	8	6	28.4	1.80	2.02	3.64
3196	Achn231381	3-ketoacyl-CoA synthase	AT1G07750	AT1G07750	45.3	637	37482	6.85	22	17	11	36.3		2.41	4.16
1092	Achn040121	70-kDa heat shock protein	HSC70-1	AT5G02500	92.9	260	71596	5.03	5	5	4	8.6	1.84		
1657	Achn040121	70-kDa heat shock protein	HSC70-1	AT5G02500	92.9	1061	71596	5.03	39	33	19	36.5	1.67		
1680	Achn040121	70-kDa heat shock protein	HSC70-1	AT5G02500	92.9	266	71596	5.03	5	4	4	10.3			1.53
2038	Achn040121	70-kDa heat shock protein	HSC70-1	AT5G02500	92.9	167	71596	5.03	3	3	3	5.2	2.05		
2080	Achn040121	70-kDa heat shock protein	HSC70-1	AT5G02500	92.9	390	71596	5.03	7	7	7	14.3			1.58
1694	Achn109261	70-kDa heat shock protein	HSC70-1	AT5G02500	93.3	139	71569	5.06	3	3	3	5.4			1.56
1346	Achn030361	Acylamino-acid-releasing enzyme	AARE	AT4G14570	67.3	171	43699	6.25	3	3	3	8.8		1.54	1.68
3847	Achn125831	Cathepsin B-like cysteine proteinase 3	RD21A	AT1G47128	57.5	244	42052	8.02	5	5	4	15.3	-1.69	-1.82	-2.54
3930	Achn125831	Cathepsin B-like cysteine proteinase 3	RD21A	AT1G47128	57.5	632	42052	8.02	32	20	10	32	-2.71	-2.38	-3.96
3727	Achn125851	Cathepsin S	RD21B	AT5G43060	57.7	143	28620	4.79	13	11	2	11	-1.62		
1274	Achn116231	Chaperone protein ClpB 1 (chaperonin ClpA/B)	CLPC1	AT5G50920	82.4	129	182879	7.12	3	3	3	2.1	1.68	2.06	1.95
1277	Achn116231	Chaperone protein ClpB 1 (chaperonin ClpA/B)	CLPC1	AT5G50920	82.4	123	182879	7.12	3	3	2	1.3	2.13	2.08	2.32
1268	Achn348721	Chaperone protein ClpB 1 (chaperonin ClpA/B)	CLPC1	AT5G50920	84.3	184	110487	8.54	4	3	3	4.4	1.78	1.78	1.88
4020	Achn281071	Proteasome subunit beta type	PBD1	AT3G22630	80.8	224	22676	5.95	7	4	4	21.1			-1.79
4052	Achn118941	Proteasome subunit beta type	PBD1	AT3G22630	84.0	227	18801	5.69	4	4	4	32			-1.84
1460	Achn010241	Protein transport protein sec23, putative	AT4G14160	AT4G14160	68.7	118	26312	6.22	2	2	2	13.1			1.54
1232	Achn244121	Putative aminopeptidase (peptidase M1, alanine aminopeptidase)	AT1G63770	AT1G63770	64.0	789	146402	5.33	18	12	10	15			1.52
1233	Achn244121	Putative aminopeptidase (peptidase M1, alanine aminopeptidase)	AT1G63770	AT1G63770	64.0	458	146402	5.33	12	8	8	9.7	1.56		1.94
1235	Achn244121	Putative aminopeptidase (peptidase M1, alanine aminopeptidase)	AT1G63770	AT1G63770	64.0	155	146402	5.33	5	2	2	3.3	1.61	1.53	2.08
1239	Achn189551	Putative aminopeptidase (Peptidase M1, alanine aminopeptidase)	AT1G63770	AT1G63770	72.3	388	99883	5.8	9	5	5	11.7			1.96
1413	Achn159241	Subtilisin-like protease	SBT1.7	AT5G67360	69.1	107	58233	5.97	2	2	2	4.2	1.71		
1580	Achn021081	Subtilisin-like protease	AT5G67090	AT5G67090	42.0	75	57595	6.6	4	3	2	5	2.17	1.59	2.23
2003	Achn027121	T-complex protein 1 subunit alpha	TCP-1	AT3G20050	73.6	197	59327	7.68	4	3	3	8.3			1.58

2004	Achn318371	T-complex protein 1 subunit alpha	TCP-1	AT3G20050	87.5	156	47172	5.53	4	3	3	8.3	1.72		1.83
2039	Achn027121	T-complex protein 1 subunit alpha	TCP-1	AT3G20050	73.6	225	59327	7.68	4	4	4	8.6			1.57
2060	Achn252581	T-complex protein 1 subunit beta	CCT2	AT5G20890	88.8	232	48540	5.48	4	3	3	8			2.00
2081	Achn252581	T-complex protein 1 subunit beta	CCT2	AT5G20890	88.8	82	48540	5.48	2	2	2	5.5	1.85	1.62	2.36
2038	Achn074011	T-complex protein 1 subunit epsilon	AT1G24510	AT1G24510	80.6	129	57980	6.12	2	2	2	4.3	2.05		
2080	Achn074011	T-complex protein 1 subunit epsilon	AT1G24510	AT1G24510	80.6	218	57980	6.12	4	4	4	8.3			1.58
2052	Achn017921	T-complex protein 1 subunit eta	AT3G11830	AT3G11830	72.7	276	51656	6.58	6	5	4	11.2	1.59		1.96
1909	Achn021851	T-complex protein 1 subunit gamma	CCT3	AT5G26360	77.0	90	76360	5.65	2	2	2	3	1.62		
1924	Achn201871	T-complex protein 1 subunit gamma	CCT3	AT5G26360	80.7	254	56256	5.47	5	3	3	11.8		1.81	2.26
2004	Achn193181	T-complex protein 1 subunit zeta	AT3G02530	AT3G02530	91.6	258	59308	5.65	6	4	4	12.2	1.72		1.83
4527	Achn073511	Ubiquitin-conjugating enzyme, putative	MMZ3	AT2G36060	91.6	137	20221	7.74	2	2	2	19	-2.31		-3.23
2308	Achn068041	Vacuolar sorting protein 4b	SKD1	AT2G27600	84.0	90	17791	7.77	2	2	2	15.6	1.98		3.15
1588	Achn200751	Xaa-pro aminopeptidase, putative (Creatinase)	APP1	AT4G36760	58.6	201	40110	5.54	4	3	3	13			1.67
1589	Achn200751	Xaa-pro aminopeptidase, putative (Creatinase)	APP1	AT4G36760	58.6	377	40110	5.54	8	6	5	21.5	1.74		2.05
1594	Achn200751	Xaa-pro aminopeptidase, putative (Creatinase)	APP1	AT4G36760	58.6	119	40110	5.54	3	3	2	7.3	1.51		1.61
Protein synthesis															
3950	Achn381821	60S ribosomal protein l9 (Ribosomal protein L6)	RPL9D	AT4G10450	80.9	147	22299	9.54	3	3	3	20.6			-2.46
2039	Achn358201	Arginine-tRNA ligase	AT4G26300	AT4G26300	67.8	124	63315	5.4	3	2	2	5.9			1.57
1707	Achn258421	ATP-binding cassette	ABCF1	AT5G60790	86.6	156	67338	6.03	4	2	2	6	1.66		1.78
1184	Achn004851	Elongation factor	LOS1	AT1G56070	93.7	374	118434	6.3	7	6	6	7.6			1.53
1196	Achn306341	Elongation factor	LOS1	AT1G56070	93.9	458	96547	6	9	8	8	13.3	1.53		1.74
1198	Achn306341	Elongation factor	LOS1	AT1G56070	93.9	243	96547	6	7	5	4	9.2	1.51		1.59
2460	Achn178351	Eukaryotic initiation factor 4A (ATP-dependent RNA helicase eIF4A)	EIF4A1	AT3G13920	93.2	455	47169	5.31	12	7	6	24.9			1.52
1268	Achn017141	Glycine-tRNA ligase	AT1G29880	AT1G29880	82.5	102	71190	6.09	2	2	2	3.7	1.78	1.78	1.88
1680	Achn017141	Glycine-tRNA ligase	AT1G29880	AT1G29880	82.5	179	71190	6.09	3	3	3	4.6			1.53
1691	Achn114311	Glycine-tRNA ligase	AT1G29880	AT1G29880	80.6	243	69315	5.96	7	5	3	8.9	1.51		
1692	Achn114311	Glycine-tRNA ligase	AT1G29880	AT1G29880	80.6	105	69315	5.96	2	2	2	4.3		1.54	1.79
Secondary metabolism															
2993	Achn326461	1-aminocyclopropane-1-carboxylate oxidase	ACO4	AT1G05010	73.9	160	35568	5.62	6	4	4	10	1.85	1.59	2.14
2911	Achn281341	Cinnamyl alcohol dehydrogenase 1	ATCAD4	AT3G19450	67.6	185	36927	5.15	5	5	4	13.4			1.65

3688	Achn226891	Polyphenol oxidase	NdhS	AT4G23890	35.4	115	65514	6.27	2	2	2	4.3			-2.48
Signal transduction															
5566	Achn262321	Calcium-binding protein, putative	AT5G47540	AT5G47540	83.3	116	54019	8.95	3	3	2	5.3	-1.54		
4452	Achn088901	Ras-related protein Rab-2-A	RABB1B	AT4G35860	81.8	159	39027	6.43	4	2	2	16			-1.70
Transporters															
3628	Achn208771	Porin/voltage-dependent anion-selective channel protein	VDAC1	AT3G01280	70.7	368	29473	7.77	7	7	6	27.5			1.71
1809	Achn217001	V-type ATP synthase alpha chain	VHA-A	AT1G78900	93.7	1386	69010	5.2	44	34	22	45.4			1.57
3532	Achn331421	V-type proton ATPase subunit E	VHA-E3	AT1G64200	73.4	236	34015	7.25	7	5	3	14.3	1.61		1.92
Unclassified															
3110	Achn166661	Extracellular calcium sensing receptor	CAS	AT5G23060	61.1	108	44357	9.54	2	2	2	5.6	1.63		
2649	Achn004491	GTPase obg	YchF1	AT1G30580	86.5	252	47515	8.06	5	4	4	14.5			1.95
3865	Achn197381	Outer envelope pore protein 24. chloroplastic	AT1G45170	AT1G45170	69.8	325	23484	9.21	5	5	5	25.9	-2.62	-1.72	-2.94
2014	Achn293111	Pro-resilin	AT5G39570	AT5G39570	41.8	194	51915	4.68	3	3	3	7.2		-1.75	
1717	Achn244961	Putative polyvinylalcohol dehydrogenase		AT1G64583	23.1	187	57463	8.6	3	3	3	8.4		3.58	
1756	Achn244961	Putative polyvinylalcohol dehydrogenase		AT1G64583	23.1	153	57463	8.6	3	2	2	7.7	3.80	6.37	14.00
1288	Achn159811	Putative uncharacterized protein P0046B10.2-1 (protein of unknown function DUF642)	AT5G11420	AT5G11420	73.7	97	43435	6.96	2	2	2	6.1		1.76	
1458	Achn159811	Putative uncharacterized protein P0046B10.2-1 (protein of unknown function DUF642)	AT5G11420	AT5G11420	73.7	178	43435	6.96	5	4	3	8.4		1.65	1.86
2759	Achn159811	Putative uncharacterized protein P0046B10.2-1 (protein of unknown function DUF642)	AT5G11420	AT5G11420	73.7	416	43435	6.96	25	20	6	23.5		1.52	1.66
2793	Achn159811	Putative uncharacterized protein P0046B10.2-1 (protein of unknown function DUF642)	AT5G11420	AT5G11420	73.7	469	43435	6.96	27	21	7	25.5		1.70	2.04
2993	Achn159811	Putative uncharacterized protein P0046B10.2-1 (protein of unknown function DUF642)	AT5G11420	AT5G11420	73.7	261	43435	6.96	6	5	4	14.8	1.85	1.59	2.14
3026	Achn159811	Putative uncharacterized protein P0046B10.2-1 (protein of unknown function DUF642)	AT5G11420	AT5G11420	73.7	207	43435	6.96	6	6	3	12			1.64
3084	Achn159811	Putative uncharacterized protein P0046B10.2-1 (protein of unknown function DUF642)	AT5G11420	AT5G11420	73.7	174	43435	6.96	4	4	4	12.5			1.87
3539	Achn182381	Rubber elongation factor	AT3G05500	AT3G05500	65.3	102	27900	6.24	2	2	2	9.1	1.73	1.74	2.73

Supplementary Tab. S4. Differentially represented proteins in harvested kiwifruit at T0-T3 that matched with homologous counterparts from the *A. thaliana* interaction network database. Data were obtained with STRING (Fig. 5).

Accession - Kiwifruit database	Protein description	Gene name (STRING)	Gene name	Accession TAIR 10	Function
Achn066661	Transitional endoplasmic reticulum ATPase, putative	AtCDC48C	AtCDC48C	AT5G03340	Cell growth/division
Achn260011	Catalase-3	CAT	CAT2	AT4G35090	Disease/defense
Achn194541	Chaperone clpb (Chaperonin ClpA/B)	HSP101	DLT1	AT1G74310	Disease/defense
Achn161991	Chaperone protein clpB (Chaperonin ClpA/B)	CLPB3	CLPB3	AT5G15450	Disease/defense
Achn340321	Chitinase	HCHIB	HCHIB	AT3G12500	Disease/defense
Achn177881	Heat shock 70 kDa protein C	BIP2	BIP2	AT5G42020	Disease/defense
Achn079561	Heat shock protein 90-2	Hsp81.4	Hsp81	AT5G56000	Disease/defense
Achn075231	Monodehydroascorbate reductase	MDAR1	MDAR1	AT3G52880	Disease/defense
Achn353051	Protein disulfide isomerase-like protein	PDIL1-1	PDIL1-1	AT1G21750	Disease/defense
Achn089541	Stress-induced-phosphoprotein	Hop3	Hop3	AT4G12400	Disease/defense
Achn052701	Superoxide dismutase [Cu-Zn]	CSD2	CSD2	AT2G28190	Disease/defense
Achn319351	Superoxide dismutase [Mn-Fe]	FSD2	FSD2	AT5G51100	Disease/defense
Achn132631	Thaumatin-like protein	OSM34	OSM34	AT4G11650	Disease/defense
Achn332061	Thaumatin-like protein	OSM34	OSM34	AT4G11650	Disease/defense
Achn132621	Thaumatin-like protein	OSM34	OSM34	AT4G11650	Disease/defense
Achn145801	Thaumatin-like protein	OSM34	OSM34	AT4G11650	Disease/defense
Achn321141	6-phosphogluconate dehydrogenase, decarboxylating	AT3G02360	AT3G02360	AT3G02360	Energy
Achn346601	6-phosphogluconolactonase	EMB2024	AT5G24400	AT5G24400	Energy
Achn079831	3-isopropylmalate dehydratase large subunit	ACO3	ACO3	AT2G05710	Energy
Achn353581	Aconitate hydratase 2	ACO3	ACO3	AT2G05710	Energy
Achn115071	Aldehyde dehydrogenase	ALDH7B4	ALDH7B4	AT1G54100	Energy
Achn022321	Aldehyde dehydrogenase	ALDH2B4	ALDH2B4	AT3G48000	Energy
Achn235801	Dihydrolipoyl dehydrogenase	mtLPD1	mtLPD1	AT1G48030	Energy
Achn354501	Enolase, putative	LOS2	LOS2	AT2G36530	Energy
Achn086741	Enolase	LOS2	LOS2	AT2G36530	Energy
Achn044851	Fructose-bisphosphate aldolase 2	FBA6	FBA6	AT2G36460	Energy
Achn067501	Fructose-bisphosphate aldolase 3	FBA2	FBA2	AT4G38970	Energy
Achn282911	Fructose-bisphosphate aldolase	PDE345	FBA3	AT2G01140	Energy
Achn014461	Glucose-6-phosphate isomerase	AT5G42740	PGIC	AT5G42740	Energy
Achn349471	Glyceraldehyde 3-phosphate dehydrogenase, putative	GAPC2	GAPC2	AT1G13440	Energy
Achn323081	Isocitrate dehydrogenase [NADP]	cICDH	CICDH	AT1G65930	Energy
Achn118341	Isocitrate dehydrogenase [NADP]	AT5G14590	AT5G14590	AT5G14590	Energy
Achn312431	Malic enzyme	NADP-ME3	NADP-ME3	AT5G25880	Energy
Achn171131	NADH-ubiquinone oxidoreductase subunit	EMB1467	AT5G37510	AT5G37510	Energy
Achn171121	NADH-ubiquinone oxidoreductase subunit	EMB1467	AT5G37510	AT5G37510	Energy
Achn239151	NADH-ubiquinone oxidoreductase subunit	EMB1467	AT5G37510	AT5G37510	Energy
Achn339391	Oxalyl-CoA decarboxylase	AT5G17380	AT5G17380	AT5G17380	Energy

Achn210541	Oxygen-evolving enhancer protein 1 of photosystem II	PSBO1	PSBO1	AT5G66570	Energy
Achn041831	Phosphoenolpyruvate carboxykinase [ATP], putative	PCK1	PCK1	AT4G37870	Energy
Achn277511	Phosphoenolpyruvate carboxylase, putative	PPC3	PEPC	AT3G14940	Energy
Achn130681	Phosphofructokinase, putative	PFK3	PFK3	AT4G26270	Energy
Achn284661	Phosphoglucomutase, putative	PGM2	PGM2	AT1G70730	Energy
Achn005301	Phosphoglycerate kinase	PGK	PGK	AT1G79550	Energy
Achn305841	Phosphoglycerate kinase	PGK	PGK	AT1G79550	Energy
Achn133211	Putative 2,3-bisphosphoglycerate-independent phosphoglycerate mutase	iPGAM1	iPGAM1	AT1G09780	Energy
Achn210351	Putative 2,3-bisphosphoglycerate-independent phosphoglycerate mutase	iPGAM1	iPGAM1	AT1G09780	Energy
Achn036401	Pyruvate decarboxylase 2	AT5G01320	PDC4	AT5G01320	Energy
Achn071161	Pyruvate kinase	AT5G08570	AT5G08570	AT5G08570	Energy
Achn008791	Ribulose bisphosphate carboxylase large chain	RBCL	RBCL	ATCG00490	Energy
Achn374161	Ribulose bisphosphate carboxylase small chain	RBCS1A	RBCS1A	AT1G67090	Energy
Achn379981	Succinate dehydrogenase subunit A	SDH1-1	SDH1-1	AT5G66760	Energy
Achn297611	Transketolase, putative	AT2G45290	TKL-2	AT2G45290	Energy
Achn215591	Transketolase, putative	AT3G60750	AT3G60750	AT3G60750	Energy
Achn340601	Triosephosphate isomerase	TIM	TIM	AT2G21170	Energy
Achn203191	Triosephosphate isomerase	TPI	TPI	AT3G55440	Energy
Achn072171	3-ketoacyl-CoA thiolase	ACAT2	ACAT2	AT5G48230	Metabolism
Achn238811	Alpha-glucan phosphorylase	PHS2	AT3G46970	AT3G46970	Metabolism
Achn224791	Cysteine synthase	OASA1	OASA1	AT4G14880	Metabolism
Achn006391	Phosphoglycerate dehydrogenase	EDA9	PGDH1	AT4G34200	Metabolism
Achn299571	D-3-phosphoglycerate dehydrogenase, putative	EDA9	PGDH1	AT4G34200	Metabolism
Achn175371	Dihydrolipoyl dehydrogenase	LPD1	LPD1	AT3G16950	Metabolism
Achn269381	Glutamine synthetase	GLN1-1	GLN1-1	AT5G37600	Metabolism
Achn348911	Glycogen debranching enzyme	ISA3	ISA3	AT4G09020	Metabolism
Achn367481	Ketol-acid reductoisomerase	AT3G58610	AT3G58610	AT3G58610	Metabolism
Achn233891	L-threonine 3-dehydrogenase	AT5G51970	SDH	AT5G51970	Metabolism
Achn090001	Ornithine aminotransferase	DELTA-OAT	DELTA-OAT	AT5G46180	Metabolism
Achn366051	Putative aldehyde dehydrogenase	ALDH12A1	ALDH12A1	AT5G62530	Metabolism
Achn182781	26S proteasome non-ATPase regulatory subunit	ATS9	RPN6	AT1G29150	Protein destination/storage
Achn026231	26S proteasome regulatory subunit	AT5G23540	RPN11	AT5G23540	Protein destination/storage
Achn040121	70-kDa heat shock protein	HSC70-1	HSC70-1	AT5G02500	Protein destination/storage
Achn109261	70-kDa heat shock protein	HSC70-1	HSC70-1	AT5G02500	Protein destination/storage
Achn116231	Chaperone protein ClpB 1 (Chaperonin ClpA/B)	CLPC1	CLPC1	AT5G50920	Protein destination/storage
Achn348721	Chaperone protein ClpB 1 (Chaperonin ClpA/B)	CLPC1	CLPC1	AT5G50920	Protein destination/storage
Achn281071	Proteasome subunit beta type	PBD1	PBD1	AT3G22630	Protein destination/storage
Achn118941	Proteasome subunit beta type	PBD1	PBD1	AT3G22630	Protein destination/storage
Achn244121	Putative aminopeptidase (peptidase M1, alanine aminopeptidase)	AT1G63770	AT1G63770	AT1G63770	Protein destination/storage
Achn189551	Putative aminopeptidase (Peptidase M1, alanine aminopeptidase)	AT1G63770	AT1G63770	AT1G63770	Protein destination/storage
Achn027121	T-complex protein 1 subunit alpha	TCP-1	TCP-1	AT3G20050	Protein destination/storage
Achn318371	T-complex protein 1 subunit alpha	TCP-1	TCP-1	AT3G20050	Protein destination/storage
Achn252581	T-complex protein 1 subunit beta	AT5G20890	CCT2	AT5G20890	Protein destination/storage

Achn074011	T-complex protein 1 subunit epsilon	AT1G24510	AT1G24510	AT1G24510	Protein destination/storage
Achn017921	T-complex protein 1 subunit eta	AT3G11830	AT3G11830	AT3G11830	Protein destination/storage
Achn021851	T-complex protein 1 subunit gamma	AT5G26360	CCT3	AT5G26360	Protein destination/storage
Achn201871	T-complex protein 1 subunit gamma	AT5G26360	CCT3	AT5G26360	Protein destination/storage
Achn193181	T-complex protein 1 subunit zeta	AT3G02530	AT3G02530	AT3G02530	Protein destination/storage
Achn200751	Xaa-pro aminopeptidase, putative (Creatinase)	APP1	APP1	AT4G36760	Protein destination/storage
Achn381821	60S ribosomal protein l9	AT4G10450	RPL9D	AT4G10450	Protein synthesis
Achn358201	Arginine-tRNA ligase	emb1027	AT4G26300	AT4G26300	Protein synthesis
Achn004851	Elongation factor	LOS1	LOS1	AT1G56070	Protein synthesis
Achn306341	Elongation factor	LOS1	LOS1	AT1G56070	Protein synthesis
Achn178351	Eukaryotic initiation factor 4A (ATP-dependent RNA helicase eIF4A)	EIF4A1	EIF4A1	AT3G13920	Protein synthesis
Achn017141	Glycine-tRNA ligase	AT1G29880	AT1G29880	AT1G29880	Protein synthesis
Achn271321	Glycine-tRNA ligase	AT1G29880	AT1G29880	AT1G29880	Protein synthesis
Achn114311	Glycine-tRNA ligase	AT1G29880	AT1G29880	AT1G29880	Protein synthesis
Achn217001	V-type ATP synthase alpha chain	VHA-A	VHA-A	AT1G78900	Transporters
Achn331421	V-type proton ATPase subunit E	VHA-E3	VHA-E3	AT1G64200	Transporters
Achn004491	GTPase obg	AT1G30580	YchF1	AT1G30580	Unclassified
Achn293111	Pro-resilin	AT5G39570	AT5G39570	AT5G39570	Unclassified

Supplementary Tab. S5. Kiwifruit allergens identified in this study as differentially represented during fruit postharvest at 4 °C. Proteins were associated with specific entries in the Allergome database (*http://www.allergome.org*) or from literature data (Bulley, 2016).

Protein	Allergene name	Allergome code	Reference
Cathepsin S - actinidin	Act d1	1	[7]
Cathepsin B-like - actinidin Act2a	Act d1	1	[7]
Thaumatin-like protein	Act d2	747	[7]
Kiwellin	Act d5	2821	[7]
Pectin methylesterase inhibitor	Act d6	3548	[7]
Pectin methylesterase	Act d7	3547	[7]
Major latex-like protein Bet VI class	Act d11	5903	[7]
Chitinase	Act d chitinase	1271	[7]

Legend to Supplementary Figures

Supplementary Fig. S1. Content of identified metabolites in kiwifruit at different postharvest stages (T0, T1, T2 and T3). Reported are metabolites identified in the corresponding hydrohalcoholic extracts.

Supplementary Fig. S2. Content of identified metabolites in kiwifruits at different postharvest stages (T0, T1, T2 and T3). Reported are metabolites identified in the corresponding organic extracts. Abbreviations: PUFA, poly-unsaturated fatty acids; DUFA, di-unsaturated fatty acids; UFA, unsaturated fatty acids; MUFA, mono-unsaturated fatty acids; SFA, saturated fatty acids; PC, phosphatidylcholine; PE, phosphatidylethanolamine; DG, digalactosyl-diacyl-glycerol.

Supplementary Fig. S3. Preparative two-dimensional gel electrophoresis of the outer pericarp proteins extracted from kiwifruit at different postharvest stages. The figure reports the results of a Sypro Ruby-stained gel loaded with a sample containing equimolar amounts of protein extracts from kiwifruit at T0, T1, T2 and T3. Preparative 2-DE and analytical 2D-DIGE were performed as reported in the experimental section. Differentially represented spots are numbered on the gel image; they were picked automatically for protein identification and analyzed for their tryptic digests by nLC-ESI-LIT-MS/MS. Protein identification results are reported in Supplementary Tables S2 and S3.

Supplementary Fig. S4. Heat-map representation of hierarchical clustering analysis of differentially represented spots (DRSs) after 2D-DIGE analysis of protein extracts from kiwifruit outer pericarp samples taken at T0-T3 stages. Hierarchical clustering analysis of DRSs was performed using Genesis 1.8.1 platform (Institute for Genomics and Bioinformatics, Graz University of Technology).

Supplementary Fig. S5. Heat-map representation of hierarchical clustering analysis of differentially represented proteins (DRPs) after combined 2D-DIGE/nLC-ESI-LIT-MS/MS analysis of protein

extracts from kiwifruit outer pericarp samples taken at T0-T3 stages. Panels A to M report proteins organized according to their functional category grouping. Hierarchical clustering analysis of DRPs was performed using Genesis 1.8.1 platform (Institute for Genomics and Bioinformatics, Graz University of Technology).

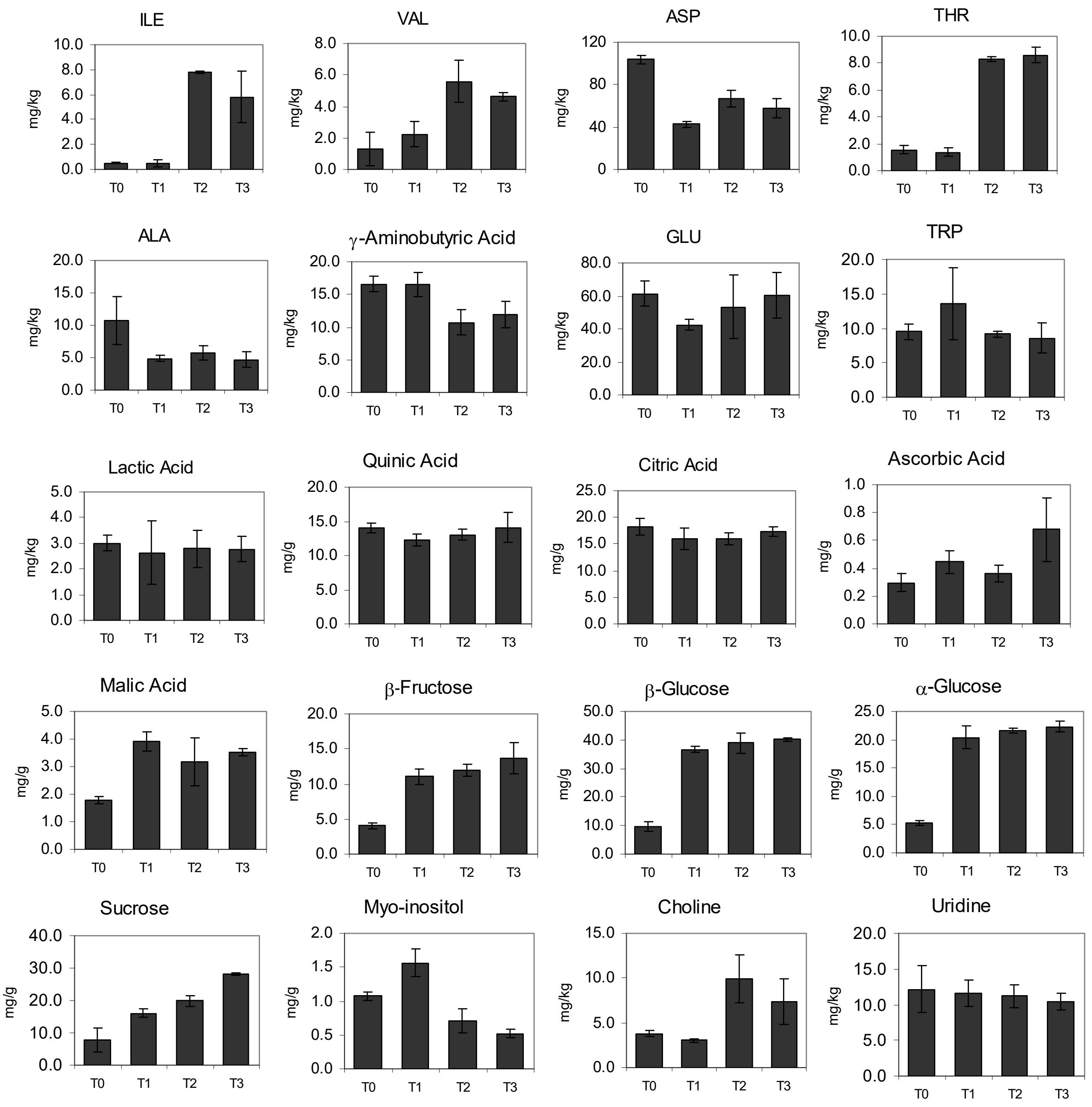

Salzano et al., Supplementary Fig. S1

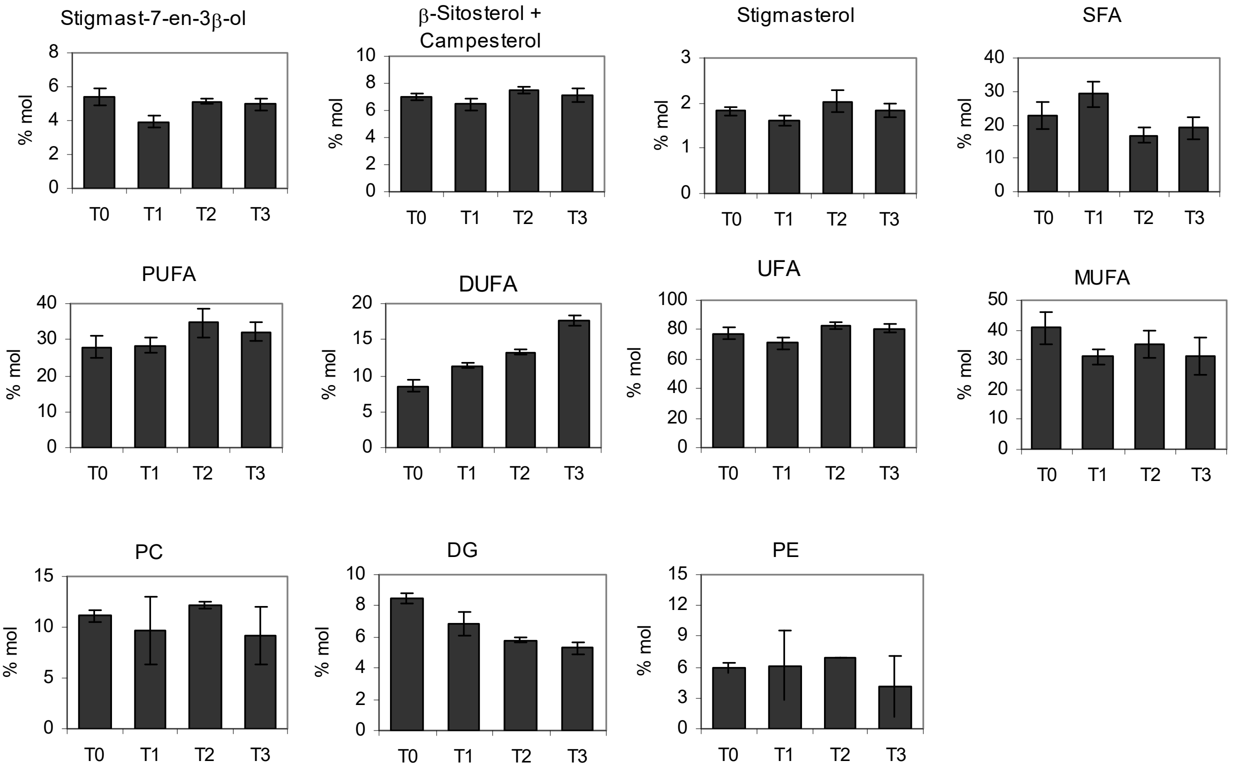

Salzano et al., Supplementary Fig. S2

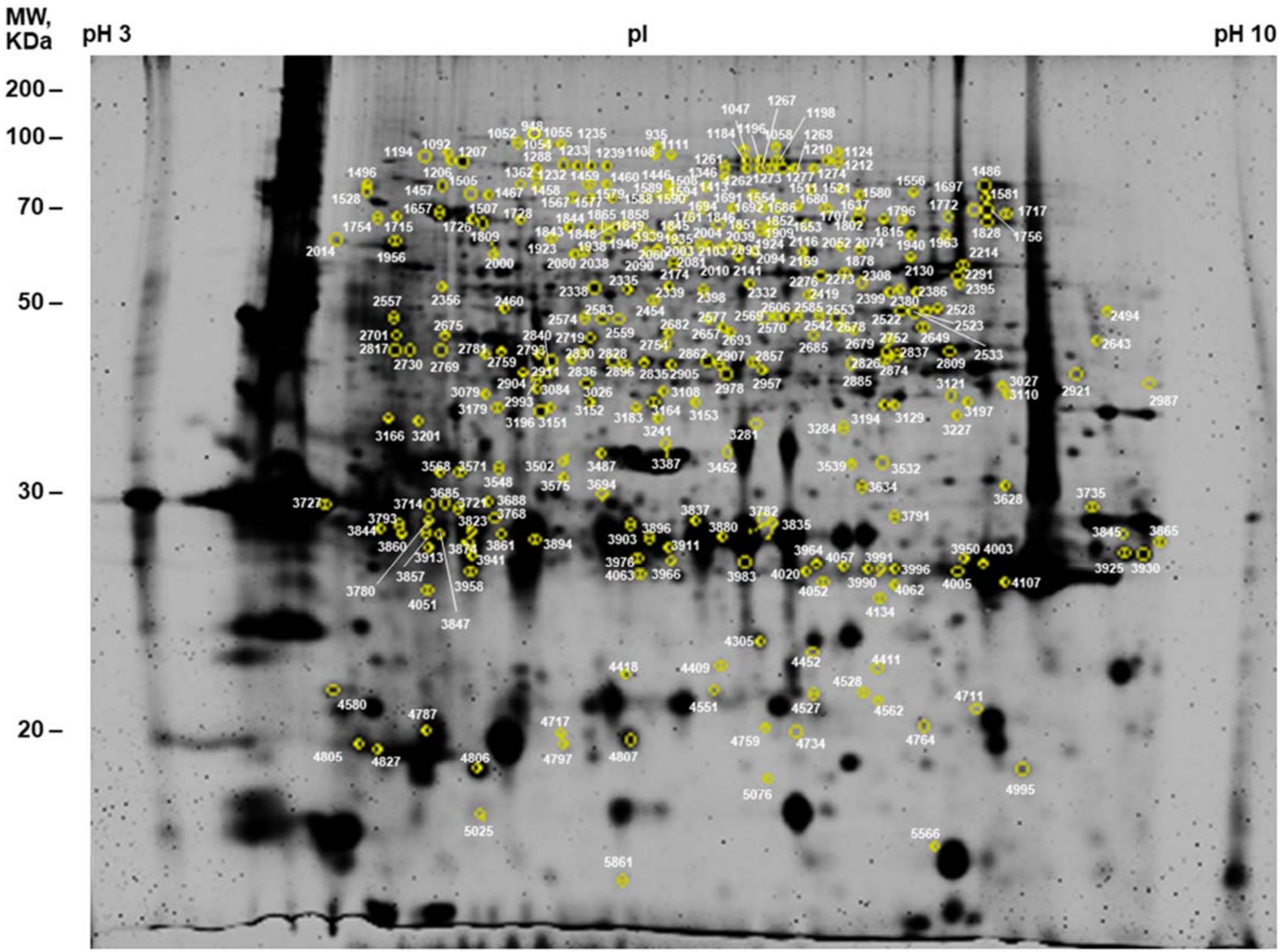

Salzano et al., Supplementary Fig. S3

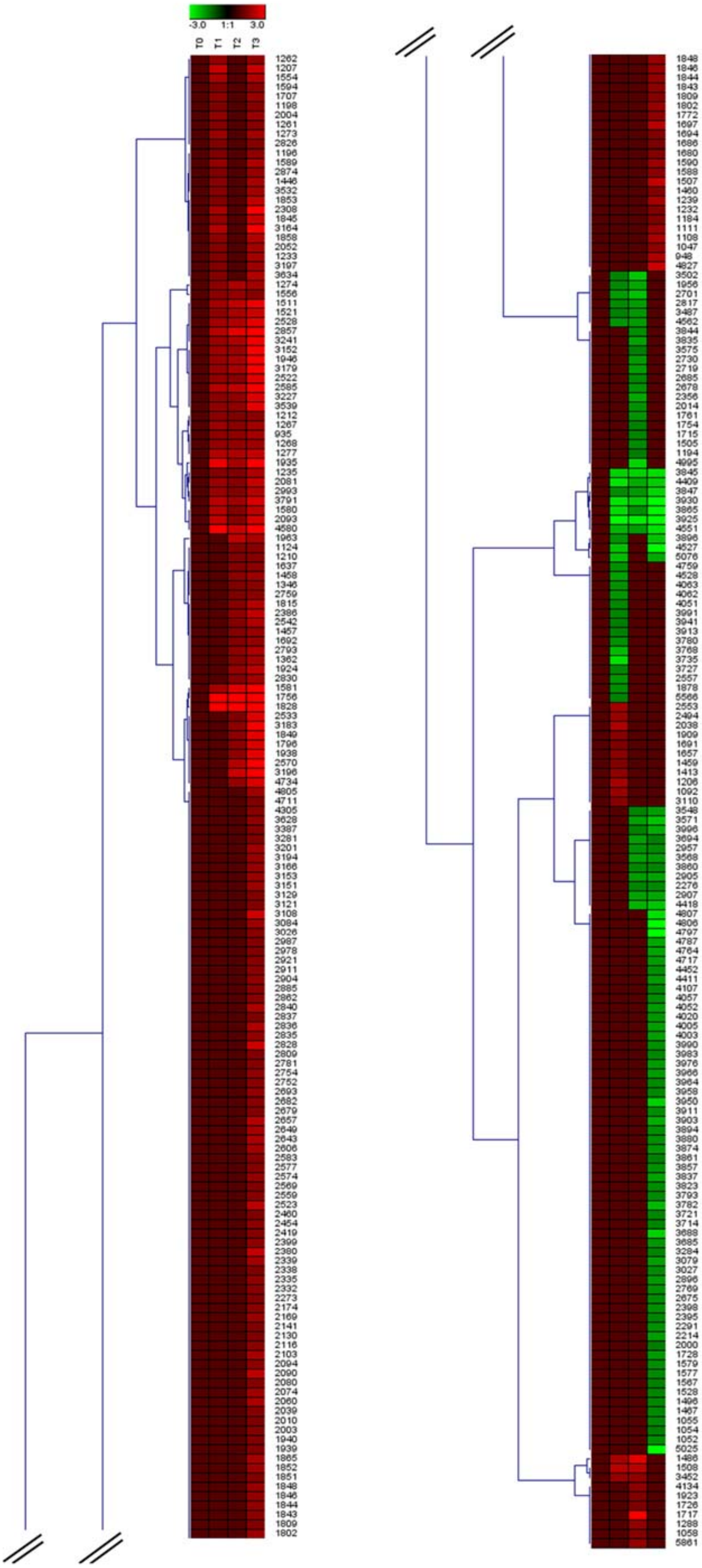

Salzano et al., Supplementary Fig. S4

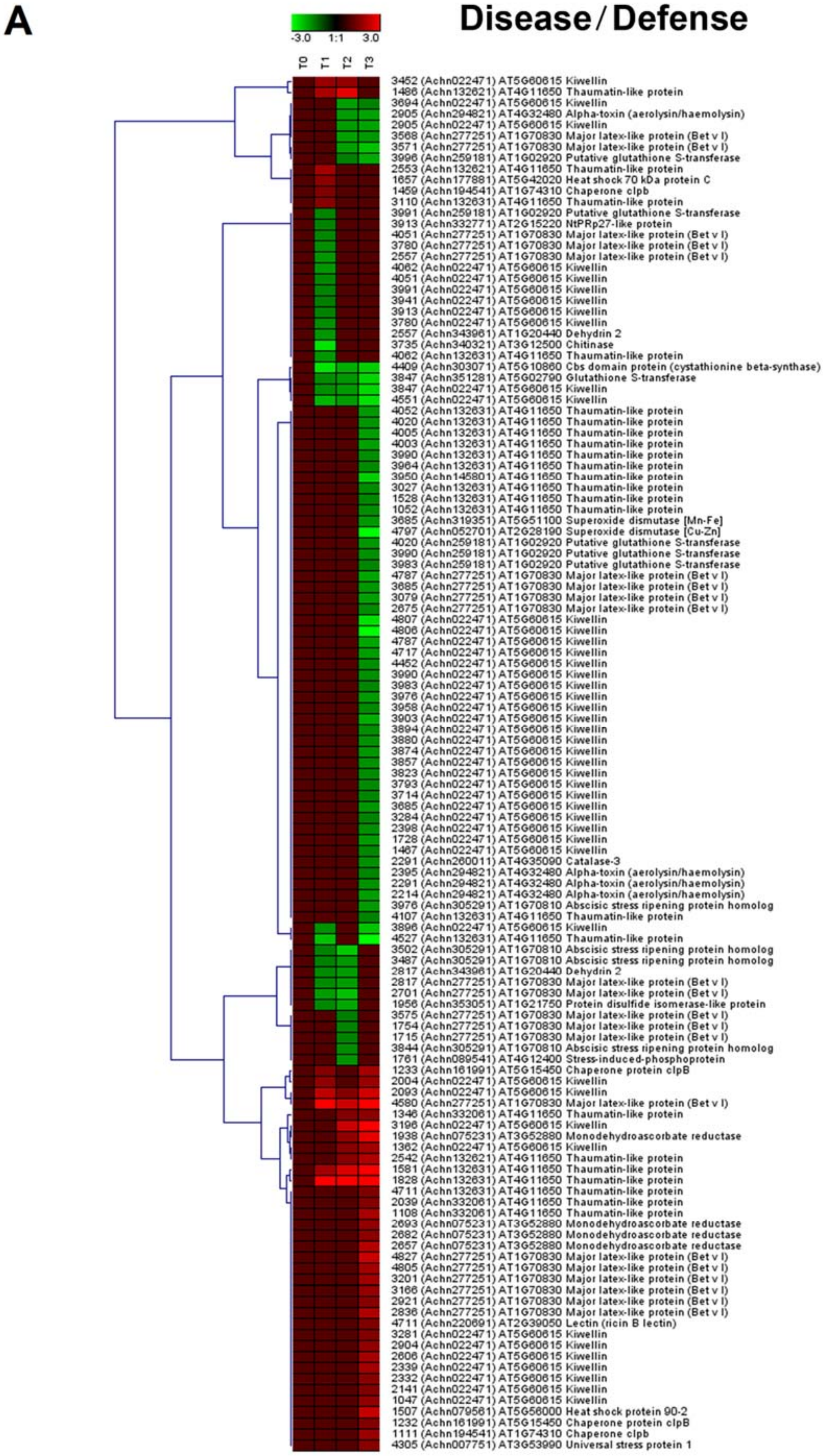

Salzano et al., Supplementary Fig. S5A

B **Protein destination and storage**

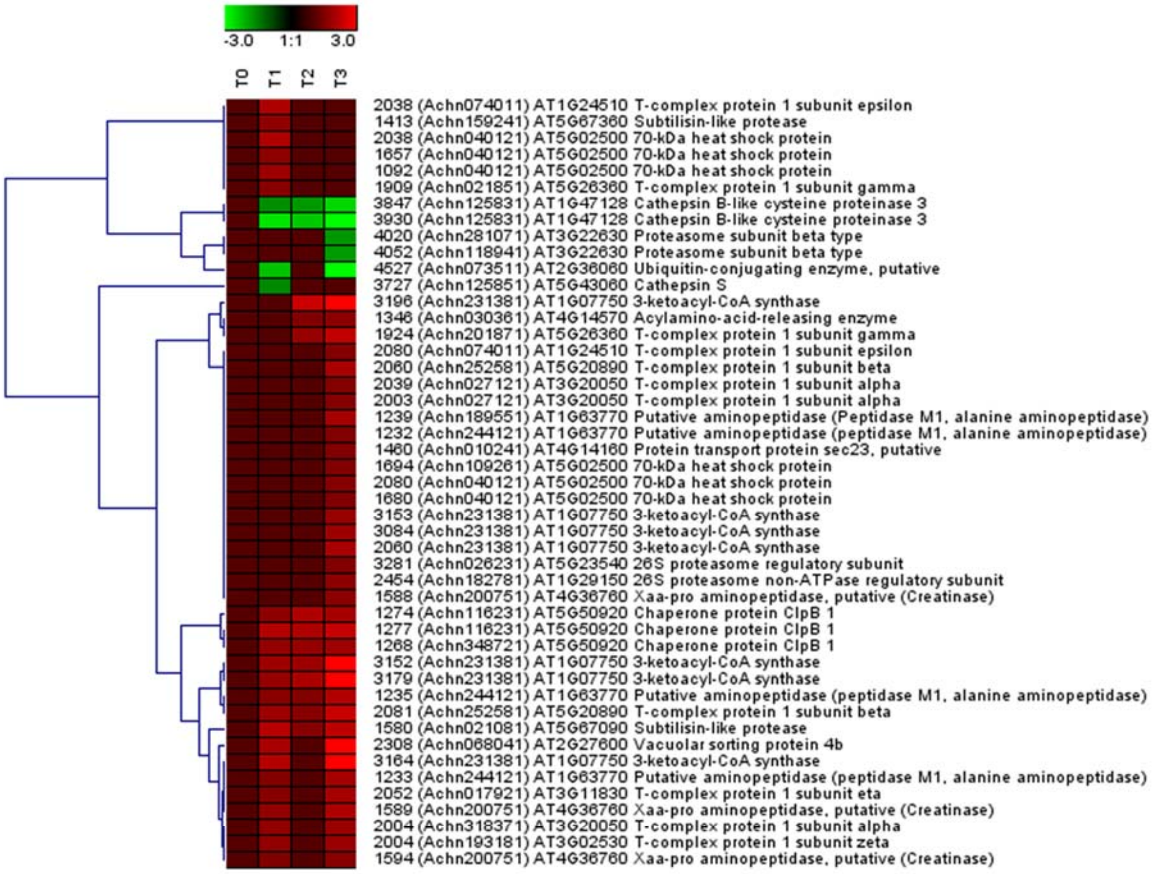

C **Protein synthesis**

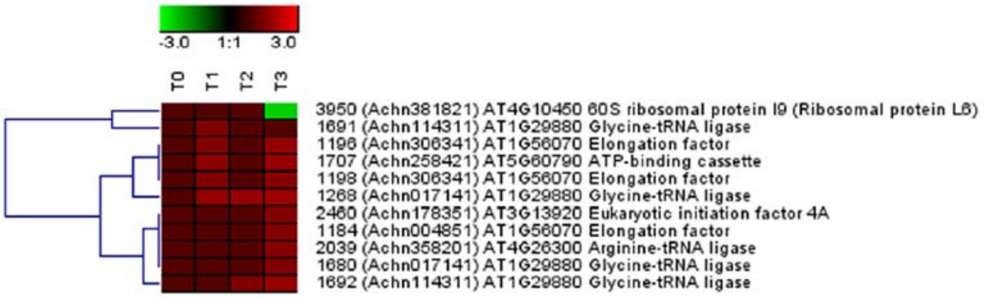

Salzano et al., Supplementary Fig. S5B, C

D

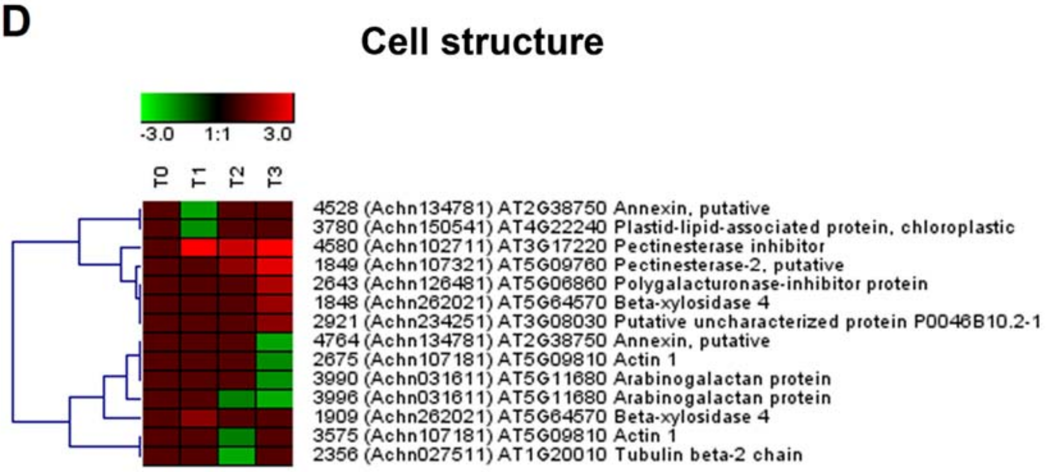

E

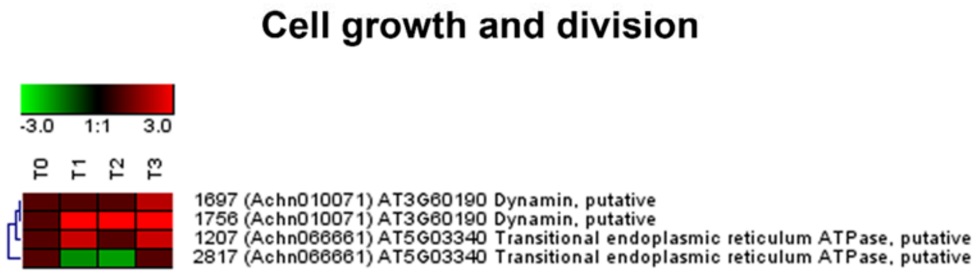

Salzano et al., Supplementary Fig. S5D, E

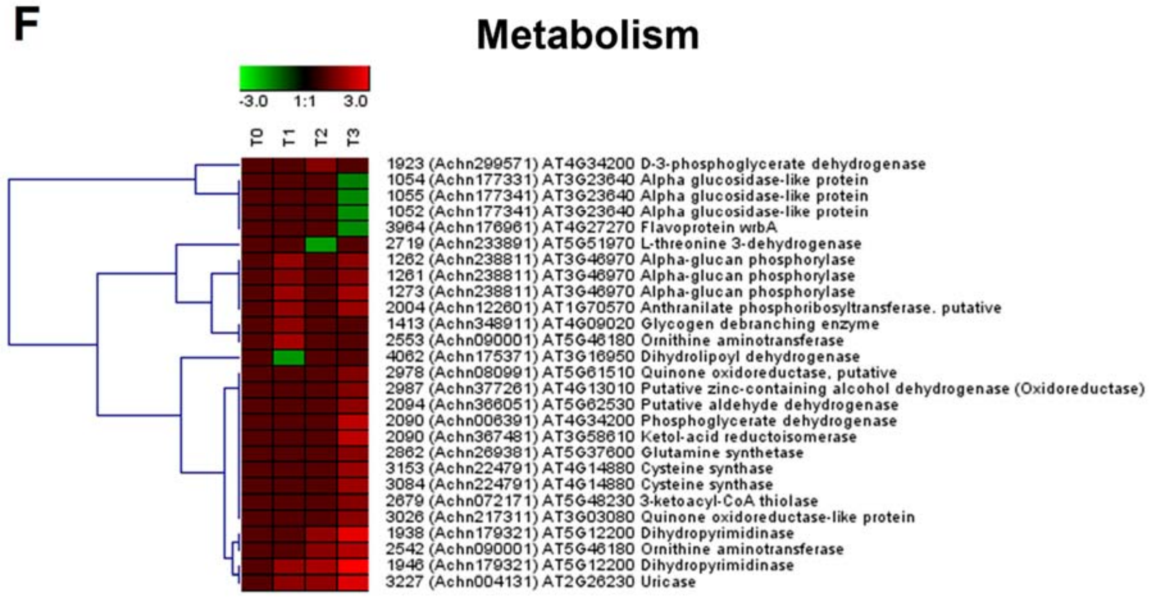

Salzano et al., Supplementary Fig. S5F

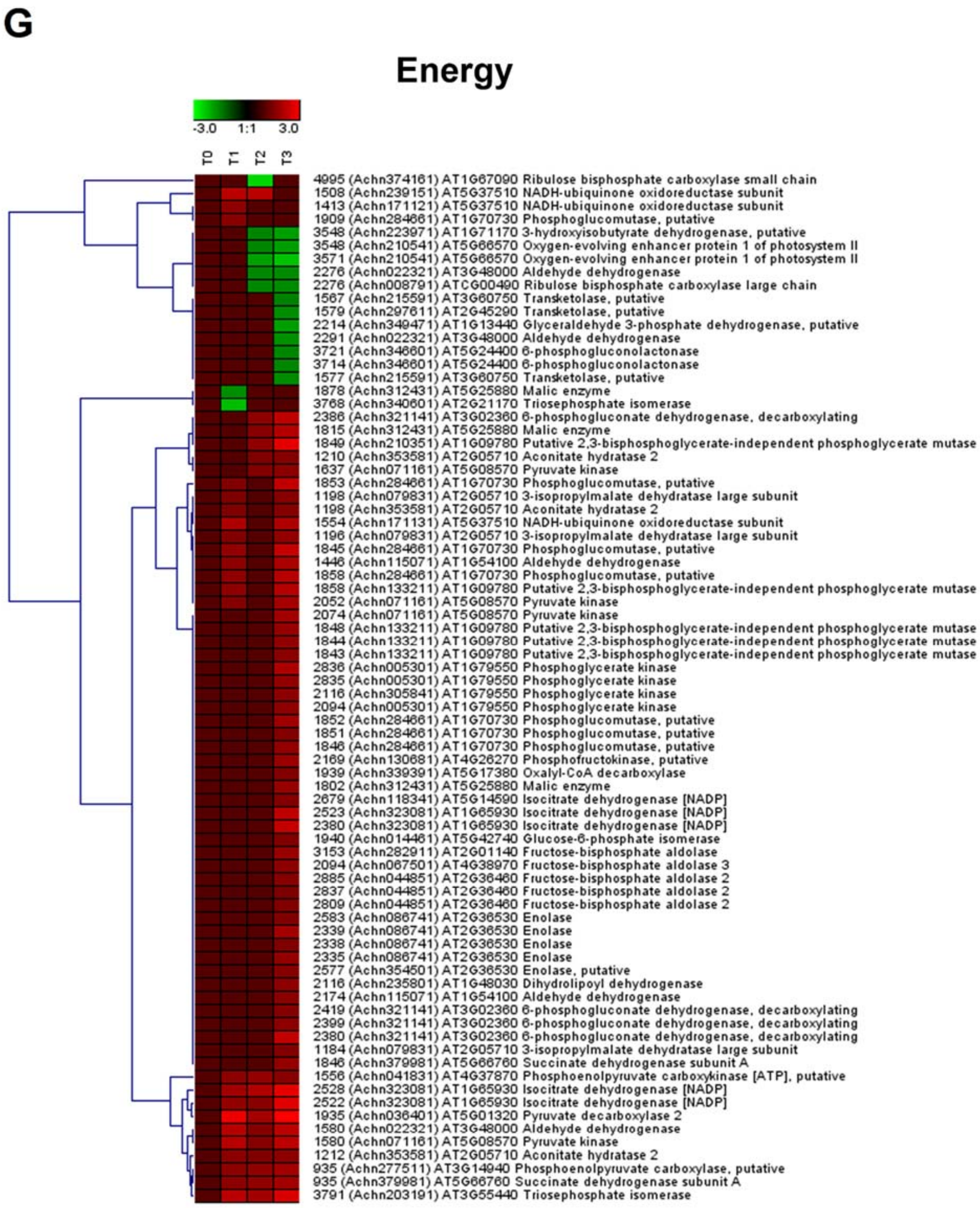

Salzano et al., Supplementary Fig. S5G

H

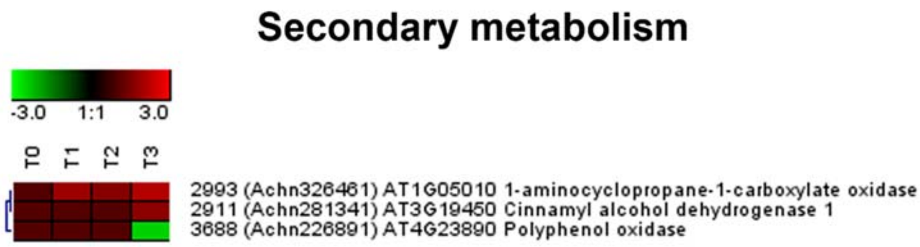

I

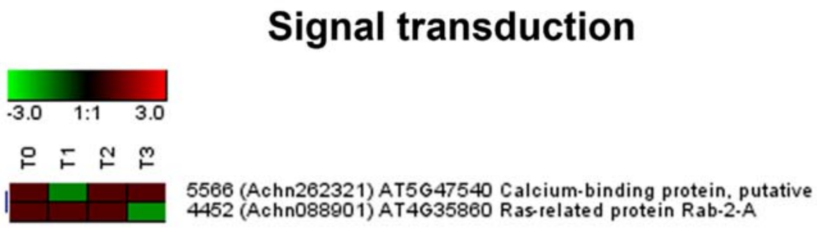

L

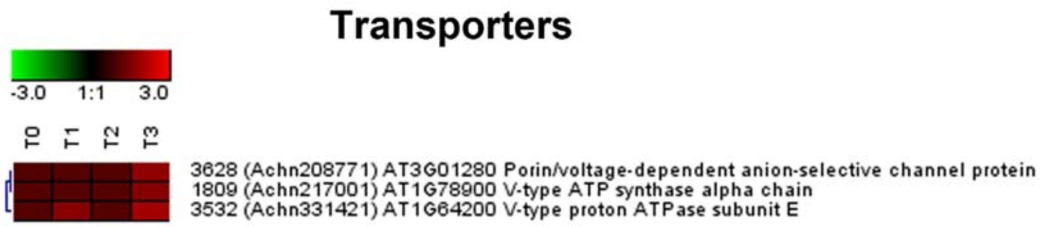

M

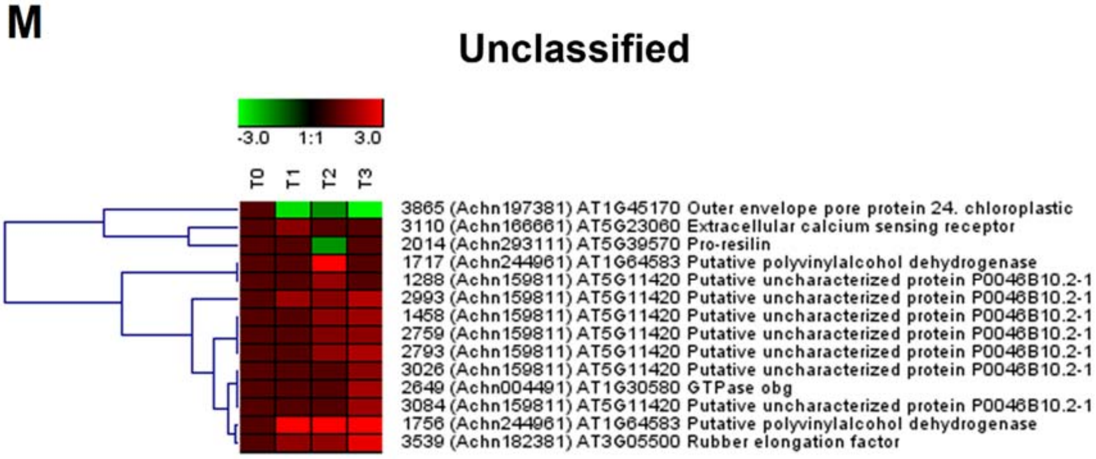

Salzano et al., Supplementary Fig. S5H, I, L, M
